# RNA-Seq in 296 phased trios provides a high-resolution map of genomic imprinting

**DOI:** 10.1186/s12915-019-0674-0

**Published:** 2019-06-24

**Authors:** Bharati Jadhav, Ramin Monajemi, Kristina K. Gagalova, Daniel Ho, Harmen H. M. Draisma, Mark A. van de Wiel, Lude Franke, Bastiaan T. Heijmans, Joyce van Meurs, Rick Jansen, GoNL Consortium, BIOS Consortium, Peter A. C. ‘t Hoen, Andrew J. Sharp, Szymon M. Kiełbasa

**Affiliations:** 1 Department of Genetics and Genomic Sciences, Hess Center for Science and Medicine, Mount Sinai School of Medicine, 1470 Madison Avenue, Room 8-116, Box 1498, New York, NY 10029 USA; 2 Department of Biomedical Data Sciences, Leiden University Medical Center, Einthovenweg 20, 2333 ZC Leiden, the Netherlands; 3 GenomeScan B.V., Plesmanlaan 1D, 2333 BZ Leiden, the Netherlands; 4 Department of Human Genetics, Leiden University Medical Center, Leiden, the Netherlands; 5 Department of Epidemiology and Biostatistics, VU University Medical Center, Amsterdam, the Netherlands; 6 Department of Genetics, University Medical Center Groningen, Groningen, the Netherlands; 7 Department of Internal Medicine, Erasmus MC, Rotterdam, the Netherlands; 8 Department of Psychiatry, VU University Medical Center, Neuroscience Campus Amsterdam, Amsterdam, the Netherlands; 9 Centre for Molecular and Biomolecular Informatics, Radboud Institute for Molecular Life Sciences, Radboud University Medical Center, Nijmegen, the Netherlands

**Keywords:** Imprinting, Allele-specific expression, Bayesian analysis, Parent-of-origin, Phased genotypes

## Abstract

**Background:**

Identification of imprinted genes, demonstrating a consistent preference towards the paternal or maternal allelic expression, is important for the understanding of gene expression regulation during embryonic development and of the molecular basis of developmental disorders with a parent-of-origin effect. Combining allelic analysis of RNA-Seq data with phased genotypes in family trios provides a powerful method to detect parent-of-origin biases in gene expression.

**Results:**

We report findings in 296 family trios from two large studies: 165 lymphoblastoid cell lines from the 1000 Genomes Project and 131 blood samples from the Genome of the Netherlands (GoNL) participants. Based on parental haplotypes, we identified > 2.8 million transcribed heterozygous SNVs phased for parental origin and developed a robust statistical framework for measuring allelic expression. We identified a total of 45 imprinted genes and one imprinted unannotated transcript, including multiple imprinted transcripts showing incomplete parental expression bias that was located adjacent to strongly imprinted genes. For example, *PXDC1*, a gene which lies adjacent to the paternally expressed gene *FAM50B*, shows a 2:1 paternal expression bias. Other imprinted genes had promoter regions that coincide with sites of parentally biased DNA methylation identified in the blood from uniparental disomy (UPD) samples, thus providing independent validation of our results. Using the stranded nature of the RNA-Seq data in lymphoblastoid cell lines, we identified multiple loci with overlapping sense/antisense transcripts, of which one is expressed paternally and the other maternally. Using a sliding window approach, we searched for imprinted expression across the entire genome, identifying a novel imprinted putative lncRNA in 13q21.2. Overall, we identified 7 transcripts showing parental bias in gene expression which were not reported in 4 other recent RNA-Seq studies of imprinting.

**Conclusions:**

Our methods and data provide a robust and high-resolution map of imprinted gene expression in the human genome.

**Electronic supplementary material:**

The online version of this article (10.1186/s12915-019-0674-0) contains supplementary material, which is available to authorized users.

## Background

Genomic imprinting is a special case of mono-allelic expression where genes are expressed in a parent-of-origin (PofO)-specific manner. Although several hypotheses exist to explain why genomic imprinting occurs, the parental conflict hypothesis [1] posits that imprinted genes evolved from a parental battle between males and females to influence the allocation of maternal resources to offspring. This type of mono-allelic expression can be observed in mammals at different developmental stages and is dependent on stage, cell, and tissue type.

Genomic imprinting plays a vital role in normal development, and errors of imprinting can underlie developmental disorders and contribute to certain cancers [2]. Imprinting significantly influences the development of cell lineages, prenatal growth, normal brain function, and metabolism [3]. Any disruption to the imprinted genes can lead to disturbed gene function and can have a deleterious effect on health. If such disruption happens at imprinted loci, it can result in imprinting disorders such as Beckwith-Wiedemann, Silver-Russell [4], Prader-Willi, and Angelman syndromes [5]; transient neonatal diabetes [6], and cancer. Wilms’ tumor, colorectal cancer, and hepatoblastoma are few examples of cancer caused due to aberrant imprinting in the *IGF2* gene [7, 8].

There are many screening methods developed and applied to discover imprinted genes such as DNA methylation, histone modification, and gene expression assays. RNA sequencing (RNA-Seq) is the most direct and comprehensive way to identify imprinted genes as it allows for quantifying relative expression of the maternal and paternal alleles (allele-specific expression or ASE) at all heterozygous sites with sufficient coverage. However, the technology is subject to several technical biases resulting in potential false positives [9]. The reference bias, caused by additional penalties in the alignment for non-reference alleles, is the most prominent of these biases [10]. Moreover, the availability of additional DNA genotype information is essential because the heterozygous sites may appear as homozygous in the RNA because of mono-allelic expression of the imprinted genes. Typically, such studies are performed without allelic inheritance information and make use of the bimodal distribution of the expression at heterozygous sites [11, 12]. This type of analysis lacks the ability to identify directionality of parental bias (i.e., assessing maternal versus paternal imprinting). Adding PofO information allows robust determination of maternal versus paternal allele-specific expression, particularly in the case of incomplete imprinting (slight bias towards the paternal or maternal allele), where bimodality in the distribution is difficult to assess. The use of PofO information is straightforward in mouse studies where reciprocal cross design is often used to identify maternal/paternal gene expression and imprinted genes [13–15]. However, in humans, assignment of parental origin requires genotype data from multiple generations. Until recently, such studies have been limited to relatively small numbers of family pedigrees [16, 17], although analyses of imprinting in much larger pedigrees have been reported recently [18, 19].

Here, we present a robust genome-wide approach to find PofO-specific gene expression and identify the signature of imprinted genes at heterozygous sites using phased DNA genotypes from parent-offspring trios and RNA-Seq data aggregated at the gene level. Our method is applied to two large-scale studies with a total of 296 trios: 165 trios from the HapMap/1000 Genomes Projects with RNA-Seq data from lymphoblastoid cell lines (LCLs) and 131 trios from the Genome-of-the-Netherlands [20]. We focus on the identification of genes and transcripts that are consistently imprinted in the population, detecting both complete imprinting (exclusive expression of the paternal or maternal allele) and incomplete imprinting (bias in expression towards the maternal or paternal allele).

## Results

We tested for imprinted gene expression using allele-specific RNA-Seq analysis of 296 parent-offspring trios derived from two independent cohorts: (i) 165 LCLs collected as part of the HapMap Project and (ii) 131 whole blood (WB) samples studied by the Genome of the Netherlands (GoNL) Consortium. In each cohort, we used phased genotypes to compute the relative expression from the maternal and paternal alleles in RNA-Seq reads at expressed heterozygous single nucleotide variants (SNVs). We analyzed 23,003 Gencode genes which had at least one heterozygous SNV with ≥ 1 overlapping RNA-Seq reads in > 10% of the samples and summed the paternal and maternal counts for all heterozygous SNVs contained in a gene, irrespective of their exonic or intronic nature. The inclusion of intronic SNVs increased the power of our test considerably despite their low individual coverage, as there were generally many more informative intronic than exonic SNVs. We applied two statistical tests to check for consistent parental expression bias of autosomal genes within the populations. The rationale for using two statistical tests, Wilcoxon signed-rank (WSR) test and ShrinkBayes (SB), is their differences in power and false-positive rate in case of low numbers of informative individuals and low expression. More details are given in Additional files 1 and 2.

**Fig. 1:**
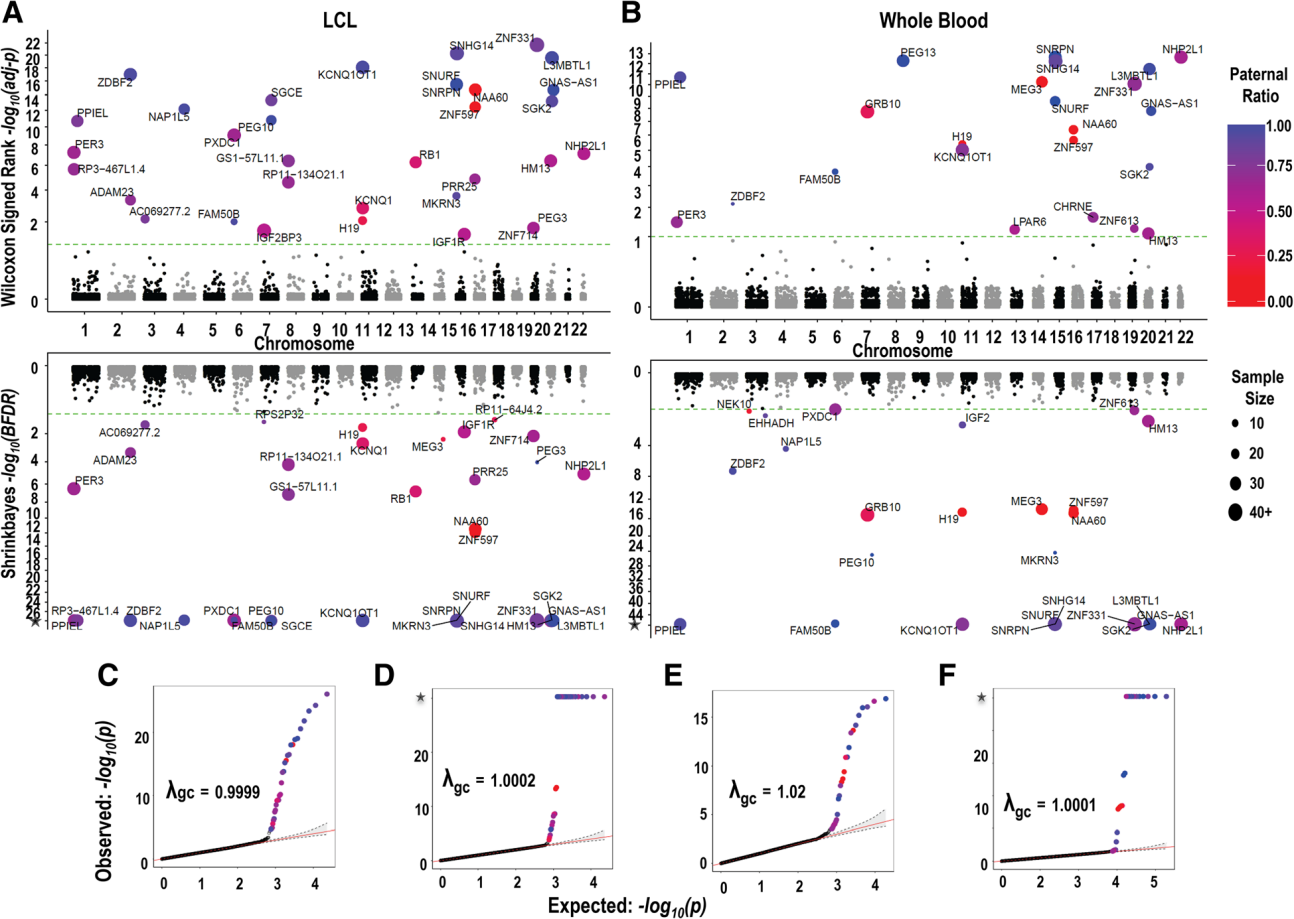
Miami and quantile-quantile plots of genome-wide results for parentally biased gene expression in 165 lymphoblastoid cell lines (LCL) and 131 whole blood (WB) samples. All data shown are based on bidirectional RNA-Seq data. In both **a** LCLs and **b** whole blood, two statistical tests for parental bias were used: the upper panel in each cohort shows the results from the paired Wilcoxon signed-rank test, and the lower panel shows the results from the *ShrinkBayes* test. −log_10_ transformed adjusted *p* values are shown on the *y*-axis and chromosome and position on the *x*-axis: the dotted green lines indicate a statistical threshold of 10% FDR, with all genes exceeding this highlighted and labeled according to their paternal expression ratio and number of informative samples (see legend). These plots show the results of the analysis based on known transcript annotations, and thus do not include the unannotated transcript at 13q21.2 identified by sliding window analysis. **c**, **e** QQ plots for the paired Wilcoxon signed-rank test in LCLs and whole blood. **d**, **f** QQ plots for *ShrinkBayes* in LCLs and whole blood. Note for *ShrinkBayes*, some of the observed –log_10_ *p* values are infinite, indicated by an asterisk on the *y*-axis. In each plot, the top 30 genes are highlighted and colored according to their paternal ratio. For both cell cohorts and statistical tests, the genomic inflation factor is approximately equal to 1. For genes with multiple UGFs (Additional file 3), we only plot data for the UGF with the most significant *p* value

**Fig. 2:**
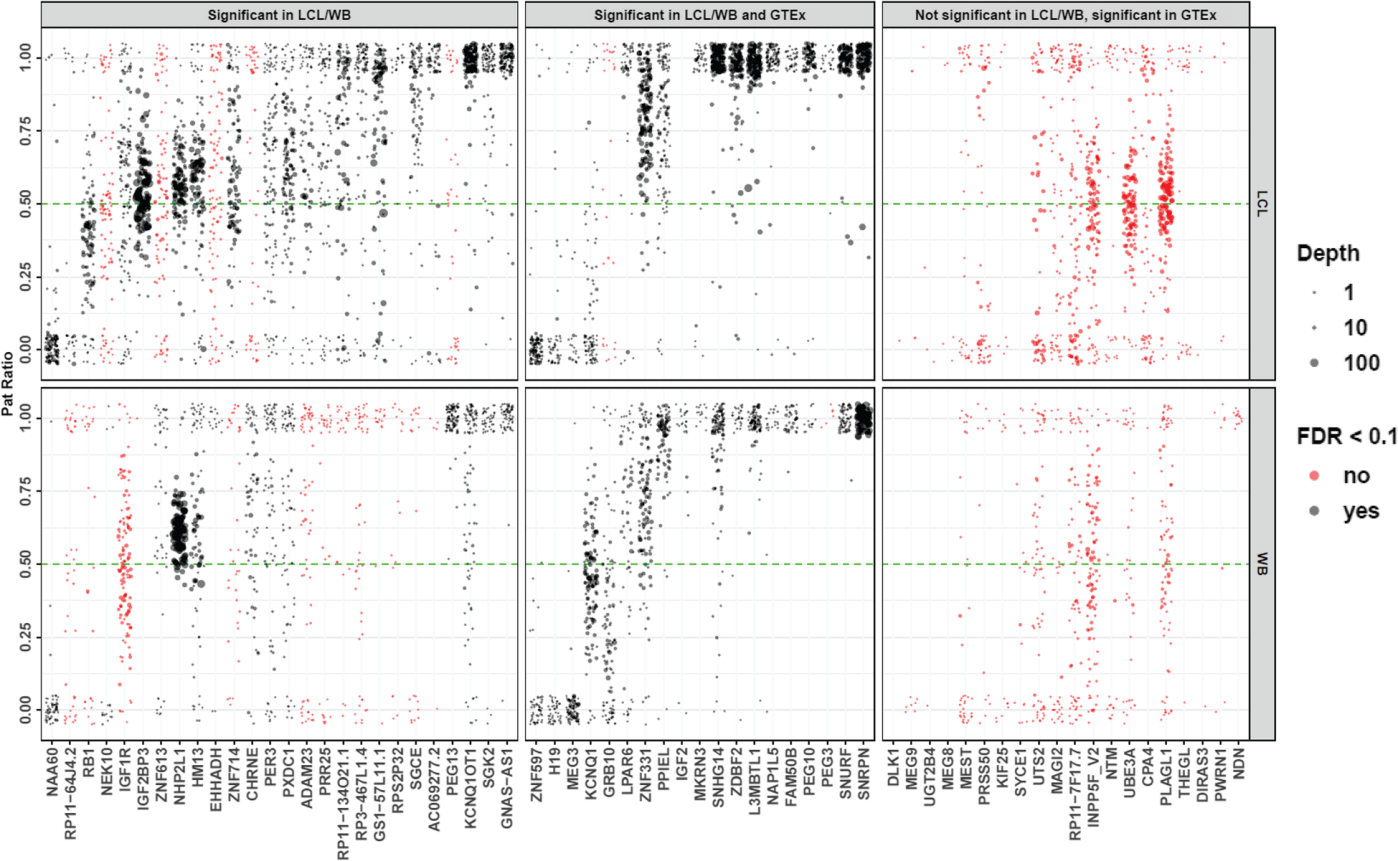
Varying degrees of parental bias among imprinted genes detected in LCLs, WB, and GTEx. Each point represents the PatRatio (the mean fraction of reads transcribed from the paternal allele) in each informative individual per gene, with the point size indicating total read depth over all heterozygous transcribed SNVs in that sample. Genes are ordered left to right by increasing mean PatRatio. The upper panel shows stranded data from LCLs, while the lower panel shows unstranded data from WB samples. Note that due to the very low read depth in some genes/individuals, several genes showed highly variable PatRatios within the population. A small *x*- and *y*-axis jitter was added to reduce overplotting effects. Genes shown in black were significant (FDR < 0.1), while those in red did not pass this statistical threshold for significance. The figure is divided into three panels: left, middle, and right panel. Genes in the middle panel were found significant in LCL and/or WB and reported as putatively imprinted in GTEx [11]; genes shown in the left panel were found significant in LCL and/or WB but not reported in GTEx; and genes shown in the right panel represent those reported as putatively imprinted in GTEx but were not identified as showing significant evidence of imprinting in either LCL or WB. Some genes in the right panel such as *DLK1*, *MEG9*, *THEGL*, *DIRAS3*, *PWRN1*, and *NDN* show evidence of parental expression bias, but the limited number of informative samples meant we did not consider these in our formal analysis. For genes with multiple UGFs (Additional file 3), we plot paternal ratios for the UGF with the most significant *p* value

Quantile-quantile plots showed a clear excess of genes with highly significant observed *p* values above the null expectation with both statistical tests and cohorts, indicating strong evidence for imprinting. Furthermore, there was no evidence of genomic inflation in our study, with all values of *λ* between 0.9999 and 1.02 (Fig. 1). To increase the resolution and avoid confounding in cases where multiple different transcripts overlapped each other, we used unique gene fragment (UGF; see the “Methods” section) annotation as the basic genomic units. Overall a total of 78 UGFs across the two populations showed significant evidence of imprinting (Additional files 3 and 4): 66 in LCLs and 43 in WB. However, the presence of overlapping transcripts, some of which were split into multiple separate annotations by our use of UGFs, created redundancy in this list. After removal of these redundancies, we further manually curated signals to (i) assign signals of imprinted expression to the gene annotation which showed the best consistency with the strand and location of data, (ii) remove transcripts where biased expression was driven by outlier samples with extreme read depth, and (iii) at loci containing multiple overlapping gene annotations, to avoid inflating the number of reported genes, we removed anonymous transcripts which appeared to represent partial gene fragments (see comments in Additional file 3). This identified a total of 45 imprinted genes across the two cohorts: 38 in LCLs and 31 in WB, with 23 identified in both populations (Fig. 1, Additional file 5: Fig. A). The paternal ratios for each of these genes in each individual are plotted in Fig. 2.

For each dataset, we classified genes as high confidence if significant (10% FDR) in both statistical tests (34 in LCLs and 19 in WB). Genes were scored as low confidence if identified as significant by a single statistical test (4 in LCLs and 11 in WB) (see Additional file 5: Fig. B and C). At 10% FDR using the paired sample Wilcoxon signed-rank (WSR) test, we found 36 and 24 significant genes in LCLs and WB, respectively. With *ShrinkBayes* (SB), we found 37 and 27 significant genes in LCLs and WB, respectively, at 10% FDR (Tables 1 and 2).

**Table 1:** High-confidence imprinted genes identified in LCLs and whole blood

	Gene name	Chr	Start (hg19)	End (hg19)	Cytogenetic band	Strand	Pat ratio (LCL_S_/LCL_U_/WB)	Preferentially expressed allele	Confidence (LCL/WB)
1	*PER3*	1	7844380	7905237	p36.23	+	0.61/0.65/0.68	Paternal	HC/LC
2	*RP3-467L1.4*	1	7870302	7887402	p36.23	–	0.81/0.69/0.62	Paternal	HC/−
3	*PPIEL*	1	39997510	40024379	p34.3	–	0.78/0.78/0.90	Paternal	HC/HC
4	*ZDBF2*	2	207139387	207179148	q33.3	+	0.94/0.94/0.95	Paternal	HC/HC
5	*ADAM23*	2	207308263	207485851	q33.3	+	0.71/0.70/0.63	Paternal	HC/−
6	*AC069277.2*	3	6532166	6777816	p26.1	+	0.79/0.79/0.80	Paternal	HC/−
7	*NAP1L5*	4	89617066	89619386	q22.1	–	0.97/0.95/0.93	Paternal	HC/LC
8	*PXDC1*	6	3722848	3752260	p25.2	–	0.65/0.64/0.68	Paternal	HC/LC
9	*FAM50B*	6	3849620	3851551	p25.2	+	0.94/0.94/1.00	Paternal	HC/HC
10	*GRB10*	7	50657760	50861159	p12.1	–	0.57/0.57/0.29	Maternal	−/HC
11	*SGCE*	7	94214542	94285521	q21.3	–	0.83/0.83/0.53	Paternal	HC/−
12	*PEG10*	7	94285637	94299007	q21.3	+	0.97/0.97/1.00	Paternal	HC/LC
13	*RP11-134O21.1*	8	2523591	2585991	p23.2	–	0.70/0.68/0.78	Paternal	HC/−
14	*GS1-57L11.1*	8	2584858	2680004	p23.2	+	0.73/0.73/0.91	Paternal	HC/−
15	*H19*	11	2016406	2022700	p15.5	–	0.10/0.26/0.00	Maternal	HC/HC
16	*KCNQ1*	11	2465914	2870339	p15.5	+	0.18/0.36/0.46	Maternal	HC/HC
17	*KCNQ1OT1*	11	2629558	2721224	p15.5	–	0.96/0.94/0.74	Paternal	HC/HC
18	*RB1*	13	48877887	49056122	q14.2	+	0.39/0.39/0.54	Maternal	HC/−
19	*LPAR6*	13	48963707	49018840	q14.2	–	0.87/0.39/0.60	Paternal	HC/LC
20	*MEG3*	14	101245747	101327368	q32.2	+	0.21/0.24/0.02	Maternal	LC/HC
21	*MKRN3*	15	23810454	23873064	q11.2	+	0.90/0.90/1.00	Paternal	HC/LC
22	*SNRPN*	15	25068794	25223870	q11.2	+	0.98/0.98/1.00	Paternal	HC/HC
23	*SNURF*	15	25200181	25245423	q11.2	+	0.98/0.98/1.00	Paternal	HC/HC
24	*SNHG14*	15	25223730	25664609	q11.2	+	0.98/0.89/0.88	Paternal	HC/HC
25	*IGF1R*	15	99192200	99507759	q26.3	+	0.56/0.56/0.50	Paternal	HC/−
26	*PRR25*	16	855443	863861	p13.3	+	0.69/0.67/0.66	Paternal	HC/−
27	*ZNF597*	16	3486104	3493542	p13.3	–	0.04/0.06/0.05	Maternal	HC/HC
28	*NAA60*	16	3493611	3536963	p13.3	+	0.06/0.05/0.03	Maternal	HC/HC
29	*ZNF714*	19	21264965	21308073	p12	+	0.62/0.62/0.63	Paternal	HC/−
30	*ZNF613*	19	52430400	52452012	q13.41	+	0.50/0.50/0.67	Paternal	−/HC
31	*ZNF331*	19	54024235	54083523	q13.42	+	0.81/0.81/0.70	Paternal	HC/HC
32	*PEG3*	19	57321445	57352096	q13.43	–	0.98/0.98/1.00	Paternal	HC/−
33	*HM13*	20	30102231	30157370	q11.21	+	0.57/0.58/0.63	Paternal	HC/HC
34	*L3MBTL1*	20	42136320	42179590	q13.12	+	0.96/0.96/0.97	Paternal	HC/HC
35	*SGK2*	20	42187608	42216877	q13.12	+	0.92/0.91/0.93	Paternal	HC/HC
36	*GNAS-AS1*	20	57393974	57425958	q13.32	–	0.96/0.96/0.98	Paternal	HC/HC
37	*NHP2L1*	22	42069934	42086508	q13.2	–	0.57/0.57/0.62	Paternal	HC/HC

High-confidence imprinted genes were classified as those transcripts showing significant evidence of parental expression bias (at 10% FDR) by both statistical tests used in at least one of the two cohorts studied. LCL_s_ and LCL_u_ indicate the results from LCL stranded and unstranded data, respectively. For genes with multiple UGFs (Additional file 3), we report paternal ratios for the UGF with the most significant *p* value

**Table 2:** Low-confidence imprinted genes identified in either LCLs or whole blood

	Gene name	Chr	Start (hg19)	End (hg19)	Cytogenetic band	Strand	Pat ratio (LCL_S_/LCL_U_/WB)	Preferentially expressed allele	Confidence (LCL/WB)
1	*NEK10*	3	27151576	27410951	p24.1	–	0.48/0.48/0.18	Maternal	−/LC
2	*EHHADH*	3	184908412	184999778	q27.2	–	0.58/0.58/0.88	Paternal	−/LC
3	*IGF2BP3*	7	23349828	23510086	p15.3	–	0.54/0.54/1.00	Paternal	LC/−
4	*RPS2P32*	7	23530092	23530983	p15.3	+	0.88/0.79/0.64	Paternal	LC/−
5	*PEG13*	8	141104993	141110634	q24.3	–	0.47/0.47/0.99	Paternal	−/LC
6	*IGF2*	11	2150342	2170833	p15.5	–	NA/ NA/0.89	Paternal	−/LC
7	*(unannotated transcript)*	13	60794418	60853802	q21.2	+	NA/0.86/NA	Paternal	LC/−
8	*RP11-64J4.2*	17	3182069	3289633	p13.3	–	0.27/0.30/0.49	Maternal	LC/−
9	*CHRNE*	17	4801069	4806369	p13.2	–	0.56/0.59/0.70	Paternal	−/LC

Low-confidence imprinted genes were classified as those transcripts showing significant evidence of parental expression bias (at 10% FDR) by just one statistical test in one of the two cohort studied. LCL_s_ and LCL_u_ indicate the results from LCL stranded and unstranded data, respectively. For genes with multiple UGFs (Additional file 3), we report paternal ratios for the UGF with the most significant *p* value

We compared the 45 imprinted genes in our dataset with those from two studies of imprinting in the Genotype-Tissue Expression (GTEx) Project [11, 12], showing that 28 were also identified as imprinted in GTEx, with one additional gene identified as “putatively imprinted” (Fig. 2, Additional file 6). In several cases, genes identified as imprinted in the GTEx cohort that we failed to replicate (e.g., *DLK1*, *MEG9*, *THEGL*, *DIRAS3*, *PWRN1*, and *NDN*) showed clear evidence of parental expression bias in our raw data, but the limited number of informative samples in our study populations meant we did not consider these in our formal analysis (Fig. 2). In all cases, we observed consistent directionality of parental bias between the two studies. Furthermore, comparison with a recent study of imprinting in a large Icelandic cohort also showed strong concordance, with 38 of the genes we identified as imprinted also observed by [19] (Additional file 6).

Using only female samples, we searched for signals of imprinting on the X chromosome. We first estimated X chromosome inactivation ratios (XCIRs) in each female, removing those samples that showed highly biased XCIR (> 80% silencing of one X chromosome), and then normalized allelic read counts for X-linked genes in each sample based on their XCIR. Analyses of these data resulted in one gene showing putative significant parental bias in LCLs (*RNA28S5*) and one gene in WB (*ARSD*). However, both were discounted as false-positive signals due to clear reference bias in both cases (Additional file 7: Fig. A-E).

### Exclusion of potential confounders

It has been reported that LCLs can sometimes undergo clonal expansion, which in turn can lead to elevated rates of mono-allelic expression [21]. As this has the potential to create artifacts that might resemble imprinting, we utilized the XCIRs we defined in females to identify and exclude LCLs with possible clonality. Focusing only on those female LCLs without skewed XCIR (XCIRs between 0.2 and 0.8, *n* = 45), we repeated the WSR test for imprinting on the 56 autosomal UGFs that had informative SNVs in at least 5 of these non-clonal LCLs. Even with this markedly reduced sample size, every gene tested showed very similar paternal ratios to those obtained in the full cohort of 165 LCLs, with 36 of the 38 (95%) genes that we report as being imprinted in LCLs achieving at least nominal significance for unequal expression of the two parental alleles (Additional file 8). Thus, we were able to exclude the possibility that artifacts due to clonality in the LCLs we studied were driving our results.

Other studies have indicated that DNA methylation can become altered during the transformation and extended culture of LCLs, raising the possibility that this might create artifacts in our LCL cohort. To assess the stability of DNA methylation at imprinted loci in LCLs, we compared published datasets of DNA methylation in LCLs and blood and compared these with methylation profiles in samples with genome-wide uniparental disomy that shows loss of imprinting (Additional file 9). This analysis showed that there was no evidence for systematic loss of imprinting in LCLs and that methylation at the differentially methylated regions of imprinted loci is broadly similar between the blood and LCLs.

### Incomplete imprinting often clusters adjacent to strongly imprinted genes

**Fig. 3:**
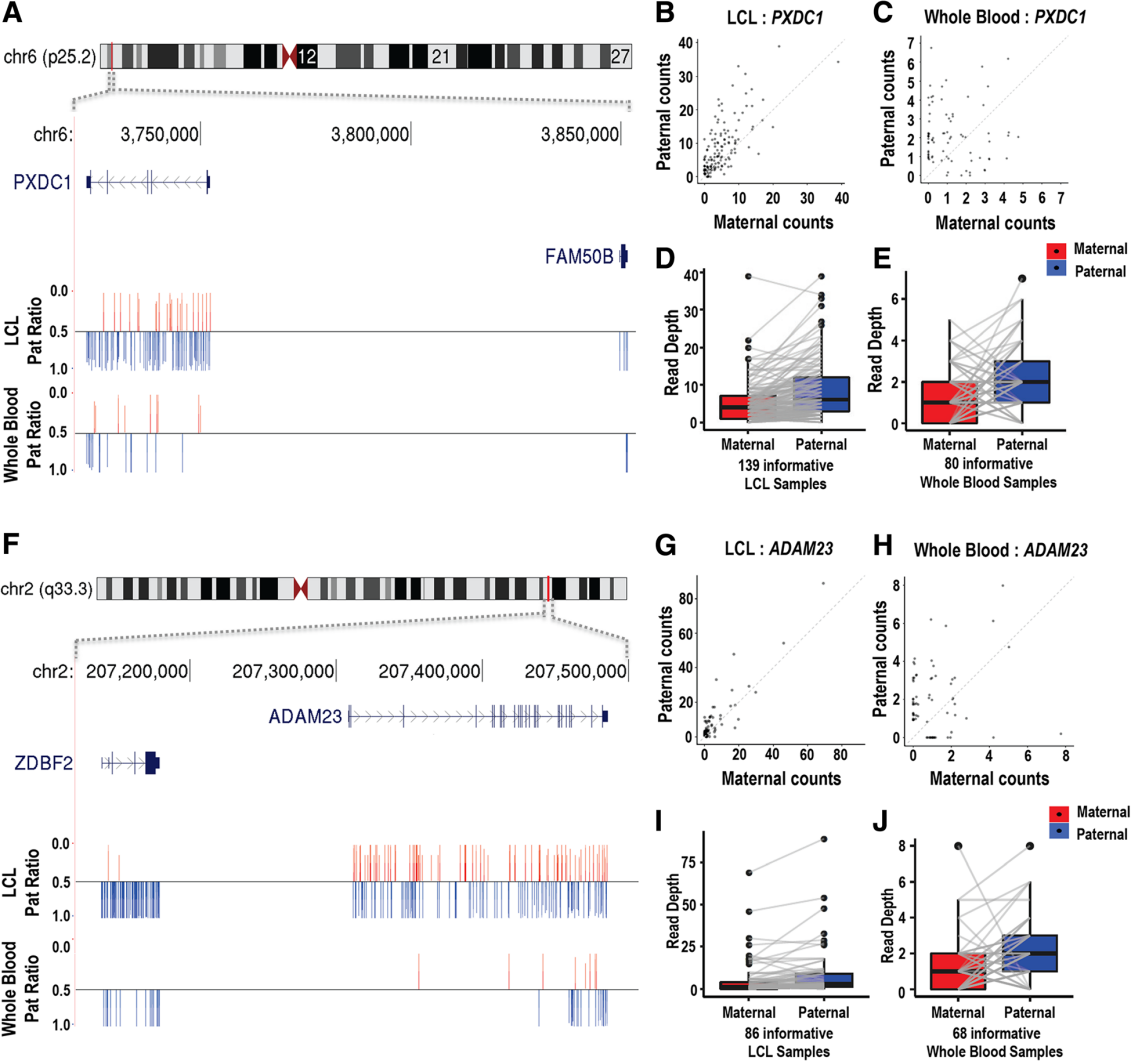
*PXDC1* and *ADAM23* are incompletely imprinted genes that lie adjacent to known imprinted genes. **a**–**e** *PXDC1* lies ~ 100 kb distal to the known paternally expressed gene *FAM50B* at 6p25.2 and, although biallelically expressed, shows approximately 2-fold higher expression from the paternal allele in both LCLs (**b**, **d**) and WB (**c**, **e**). **f**–**j** *ADAM23* lies ~ 130 kb distal to the known paternally expressed gene *ZDBF2* at 2q33.3 and also exhibits ~ 2-fold over-expression from the paternal allele in LCLs (**g**, **i**) and WB (**h**, **j**). **a**, **f** The mean fraction of reads transcribed from the paternal allele at every informative SNV position (the Pat ratio) is shown as bar, using a baseline of 0.5 (corresponding to equal expression of the two parental alleles). SNVs with preferential paternal expression (Pat ratio > 0.5) are shown in blue, while SNVs with preferential maternal expression (Pat ratio < 0.5) are shown in red. **d**/**e**, **i**/**j** Vectors join the allelic expression values within each informative individual based on the sum of total RNA-Seq reads overlapping phased heterozygous SNVs within each gene

Most previous studies have identified imprinted genes based on the complete silencing of one parental allele. However, our large population sample and the quantitative nature of our assay identified several genes with biallelic expression, but which showed a significant bias for increased expression of one of the two parental alleles (Fig. 2). In many cases, these incompletely imprinted genes occurred in close proximity to previously known imprinted genes that show mono-allelic expression. For example, we identified *PXDC1*, which lies ~ 100 kb distal to the known imprinted *FAM50B* at 6p25.2, as showing a 2:1 paternal expression bias (*PXDC1*, paternal ratio of 0.65 and 0.68 in LCL and WB, respectively) (Fig. 3), in line with recently published studies [18, 19]. Similarly, *ADAM23*, which lies ~ 130 kb distal to *ZDBF2* at 2q33.3, also exhibits ~ 2-fold over-expression from the paternal allele (*ADAM23*, paternal ratio of 0.71 in LCL), consistent with previous reports in both humans and mice [15, 16, 22]. Overall, we identified 11 clusters of imprinted genes (defined here as two or more imprinted genes separated by < 500 kb), with 25 of the 45 imprinted genes we report located in these clusters. Using published datasets of imprinted DNA methylation [19, 23, 24], we observed that in several cases, genes with incomplete imprinting lie in close proximity to the regions with parental-specific methylation marks, providing independent support for imprinting at these loci (Additional file 10). Notable examples include *PRR25* (paternal ratio = 0.69 in LCLs) and the overlapping transcripts *PER3*/*RP3-467L1.4* (paternal ratio = 0.61 and 0.81, respectively, in LCLs, shown in Additional file 11). A recent study [19] identified that both *PRR25* and *PER3* overlap CpG islands showing preferential maternal methylation. While Zink et al. did identify *PER3* and *RP3-467L1.4* as showing parental expression bias, it was not reported that the *PRR25* gene itself was imprinted. Thus, our data suggest *PRR25* is a novel incompletely imprinted gene.

To systematically investigate whether weaker imprinting localizes around strongly imprinted genes, we used data from a sliding window analysis across the genome in LCLs (detailed below) to test for enrichment of parental expression bias around known imprinted genes. Here, we choose a bin size of 25 kb, as this is approximately midway between the median gene size (~ 30 kb) and the median UGF size (~ 20 kb). Within each bin, we aggregated maternal and paternal read counts for all available heterozygous SNVs and calculated the WSR *p* value for parental expression bias for each 25 kb window. We took the set of all 25 kb non-overlapping windows that lie within ± 250 kb of strongly imprinted genes (those with paternal ratios ≤ 0.1 or ≥ 0.9), removing any windows that overlapped other strongly imprinted genes, and compared the *p* values for parental expression bias in the resulting set of 175 25 kb windows versus all 25 kb windows in the rest of the genome (*n* = 58,951). We observed that regions surrounding the strongly imprinted genes are significantly enriched for signals of parental expression bias (permutation *p* = 0.0005). Thus, our observations extend the known clustering of imprinted genes in the mammalian genome, showing that the effects of genomic imprinting can extend over broad regions and cause genes to show differing extents of parentally biased expression.

**Fig. 4:**
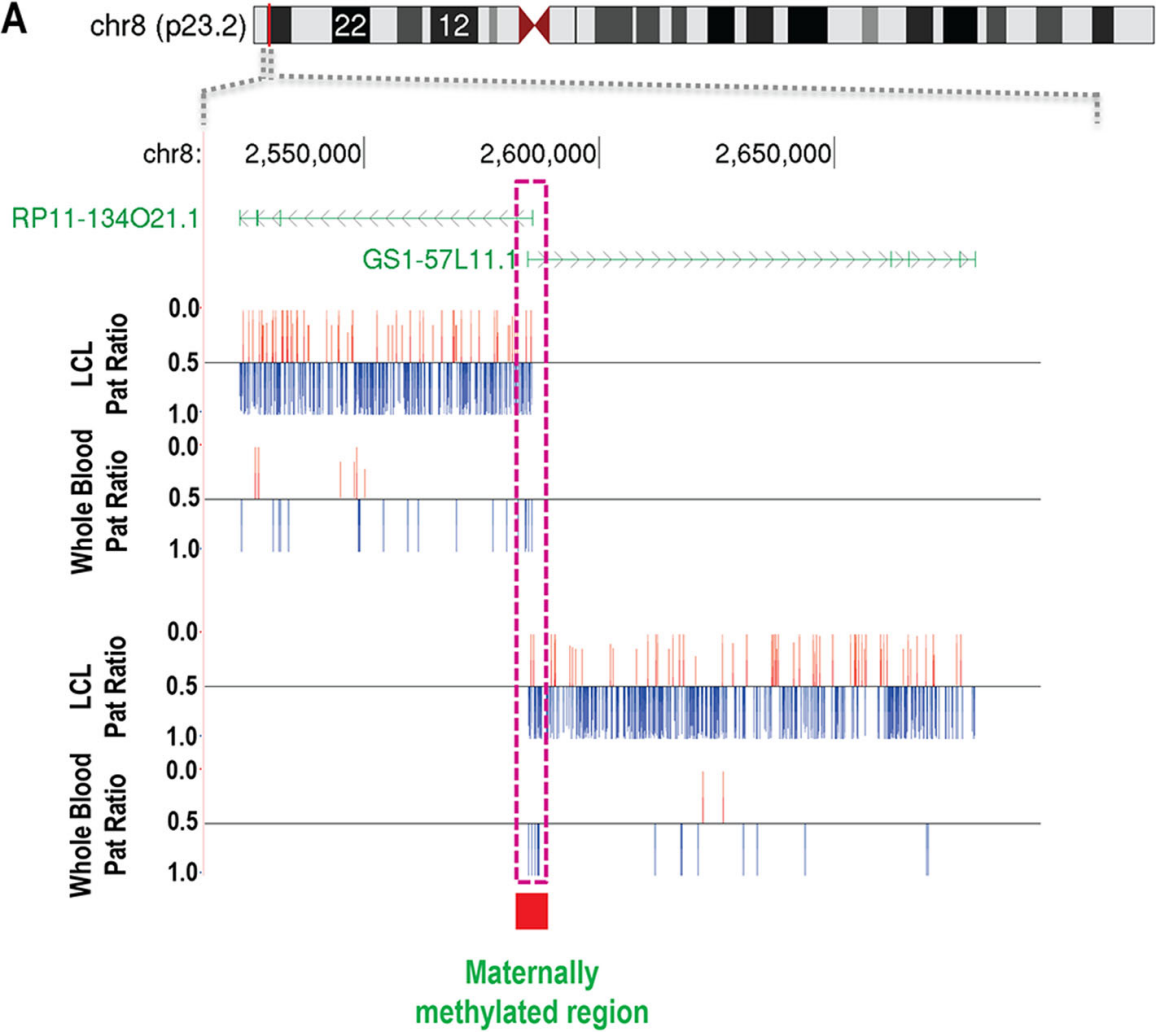
Two imprinted transcripts located at 8p23.2 share a bidirectional promoter that coincides with a maternally methylated locus. *RP11-134O21.1* and *GS1-57L11.1* are expressed from opposing strands, and both show ~ 2-fold expression from the paternal versus maternal allele in LCLs. Prior DNA methylation studies [25] identified a region of increased maternal methylation located at the shared promoter of these two transcripts, confirming parent-of-origin effects at this locus, and indicating this as the likely regulatory element controlling imprinted expression at this locus

In another example, we identified two anonymous transcripts *RP11-134O21.1* and *GS1-57L11.1* at 8p23.2 as showing a ~ 2:1 preferential expression of the paternal allele (Fig. 4). Consistent with our observations, *RP11-134O21.1* has been previously reported as showing signs suggestive of imprinting [11]. Additionally, our previous studies of blood samples from patients with uniparental disomy (UPD) [25] identified a maternally methylated region located at the bidirectional promoter of these two transcripts, thus providing independent validation of our results.

### Strand-specific RNA-Seq data provides improved resolution in cases of overlapping sense/antisense genes

**Fig. 5:**
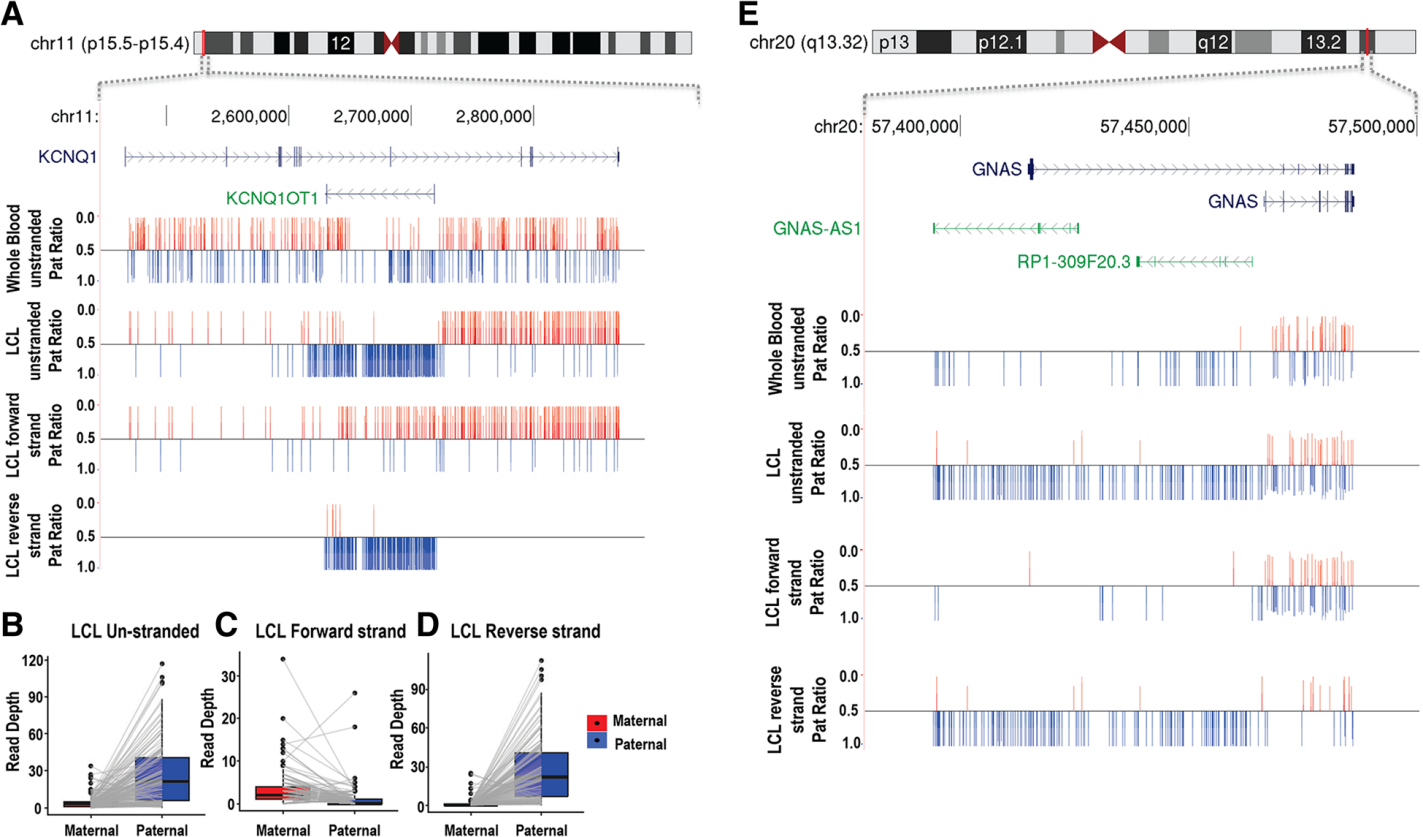
Stranded RNA-Seq data provides improved resolution of imprinting at overlapping antisense genes. Several loci in the genome contain multiple imprinted transcripts, including pairs of overlapping antisense genes with opposite imprinting patterns. Strand-specific RNA-Seq provided considerably improved ability to discern the correct imprinting patterns at these loci when compared to the use of unstranded libraries. **a**–**d** *KCNQ1* and *KCNQ1OT1* lie within the 11p15.5 imprinted region. *KCNQ1* on the plus strand is maternally expressed, while *KCNQ1OT1* on the negative strand is paternally expressed. In whole blood where only unstranded data was available, no significant parental bias was detected from either transcript, likely due to the combined signal from the two overlapping transcripts giving the appearance of biparental expression. However, the use of stranded RNA-Seq in LCLs clearly shows that the two transcripts are antisense and have opposite imprinting patterns. **e** Similarly, *GNAS* and *GNAS-AS1* are antisense transcripts located in 20q13.32. In LCLs, the stranded RNA-Seq data shows that while *GNAS-AS1* is a paternally expressed imprinted gene, *GNAS* shows biparental expression

In LCLs, the availability of strand-specific RNA-Seq data allowed the quantification of maternal and paternal counts from the forward and reverse strands separately. In the majority of cases, the results obtained using stranded data were very similar to those obtained when aggregate data from both strands were considered. However, at the loci where overlapping genes were transcribed from both forward and reverse strands, the results gained using unstranded RNA-Seq sometimes yielded misleading results that differed from those obtained using stranded data. For *KCNQ1*/*KCNQ1OT1*, *RB1*/*LPAR6*, *BMP8A/PPIEL-RP11-69E11.4*, and *PER3*/*RP3-467L1.4*, only the use of strand-specific data was able to unambiguously determine the correct imprinting status of these genes (Fig. 5). Consistent with prior studies of these loci, strand-specific data demonstrated that several sense and antisense transcript pairs displayed opposite parental bias: well-known examples of such scenario are *KCNQ1* which is maternally expressed, whereas *KCNQ1OT1* is paternally expressed [26]. Another example is *RB1*, which is maternally expressed, whereas *LPAR6* is paternally expressed (Fig. 5 and Table 1).

### Imprinting patterns at the loci with multiple isoforms and overlapping transcripts

Previous studies have noted complex patterns of imprinting at certain genomic loci, such as isoform-specific imprinting, or imprinted genes that overlap with other non-imprinted genes [24]. Using data from the location of individual informative SNVs within the imprinted genes we report, we identified several loci that exhibited differential imprinting patterns among subregions of gene annotations.

**Fig. 6:**
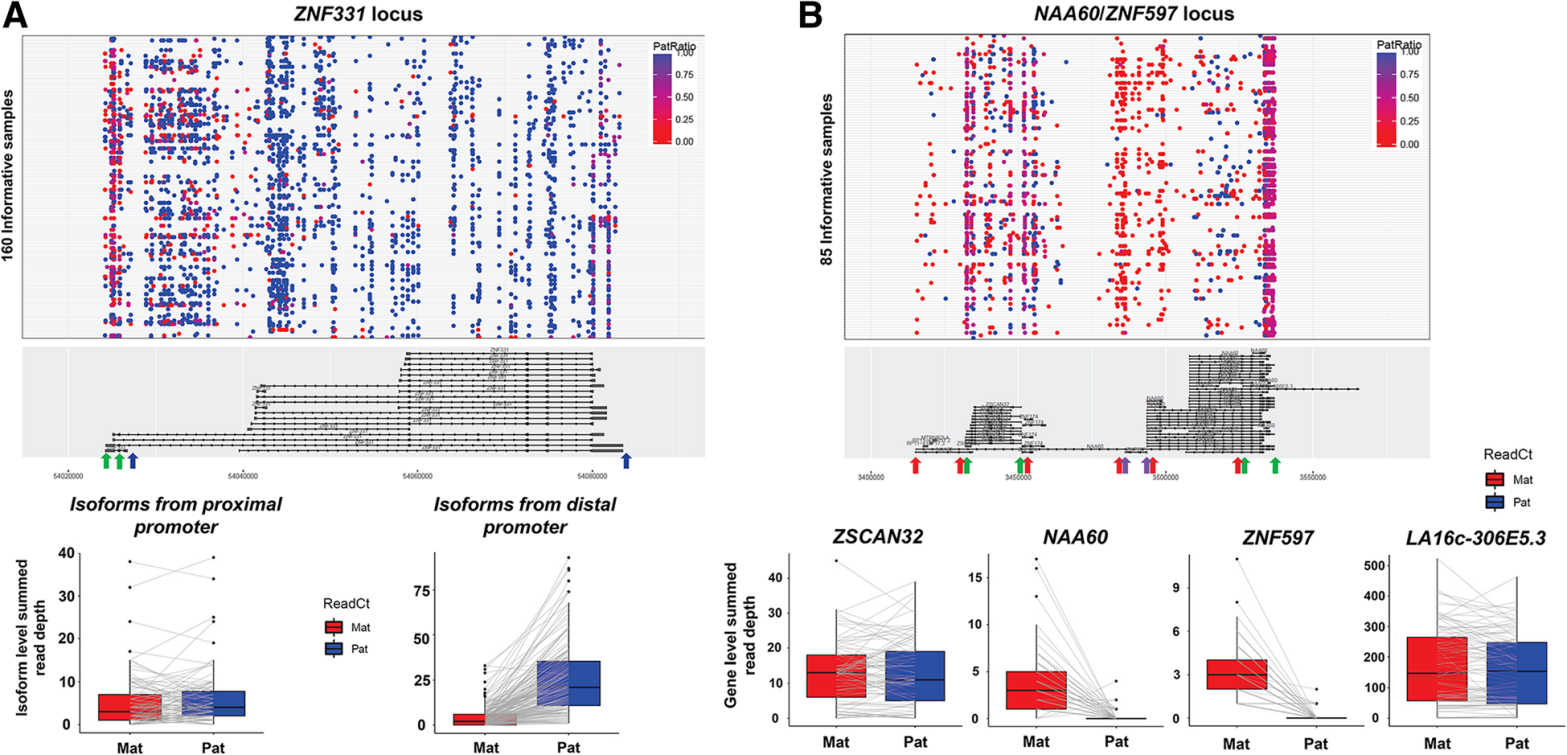
Complex patterns of imprinting at the *ZNF331* and *NAA60*/*ZNF597* loci revealed by phasing hundreds of transcribed SNVs. **a** Isoform-specific imprinting of *ZNF331* has been previously reported [24, 27] where the longer isoform has biallelic expression in human LCLs, while the shorter isoforms have paternal expression. Isoforms expressed from the proximal promoter (boundaries indicated by green arrows under gene plot) show biallelic expression (left boxplot), while longer isoforms of the gene (boundaries indicated by the blue arrows) show strong paternal expression bias (right boxplot). Thus, depending on the position of the observed heterozygous SNVs within the *ZNF331* gene, an individual may show different patterns of allelic bias. **b** Parental expression bias at *NAA60/ZNF597*, a complex locus that contains multiple overlapping imprinted and non-imprinted genes. The longest annotated isoform of *NAA60* overlaps the imprinted gene *ZNF597*, and also the biallelically expressed genes, *ZSCAN32*, *ZNF174*, and *LA16c-306E5.3*. Considering SNVs within the boundaries of *ZSCAN32* and *LA16c-306E5.3* (regions defined by the green arrows) yields no evidence of imprinted expression, even though these are also contained within the longest annotated isoform of *NAA60*. However, considering SNVs located within the UGFs that uniquely describe *ZNF597* (defined by red arrows) or *NAA60* (defined by purple arrows) reveals almost exclusive expression from the maternal allele for these two genes. In the upper gene plots, each dot represents a single heterozygous SNV, which are colored to indicate the allelic ratio of the overlapping reads. Box plots show aggregate maternal and paternal read counts per individual

One example of this phenomenon is *ZNF331*, which has multiple different isoforms with different transcription start sites. As shown in Fig. 6, isoforms of *ZNF331* that start at the most proximal promoter show no evidence of imprinting, while other isoforms transcribed from more distal promoters show ~ 90% expression from the paternal allele. Previous reports [24] have suggested that in the blood leukocytes, there is maternal-specific expression from the most proximal promoter of *ZNF331*, while our analysis indicates that in LCLs, these isoforms show equal biparental expression.

The data for *HM13* suggest that this may show isoform-specific imprinting, with the longest isoforms showing a strong paternal expression bias, while shorter isoforms are biparentally expressed in the blood. An alternative possibility is that there is a parent-of-origin sensitive use of the most distal of the alternative polyadenylation sites in *HM13* as a consequence of the imprinting of the *MCTS2* gene, similar to what is observed in mice [28, 29] (Additional file 12).

Another example of similar complexity is the *NAA60/ZNF597* locus, which from prior studies is known to show isoform- and cell type-specific imprinting [16, 30]. Additionally, this locus contains multiple overlapping transcripts, only some of which are imprinted. The longest isoform of *NAA60* (forward strand) overlaps several other genes on the same or opposite strand, including *ZNF174*, *ZSCAN32 LA16c-306E5.3*, and *MTRNR2L4*. With strand-specific data and UGF annotations, we observed that the SNVs that overlap either *ZSCAN32* (reverse strand), *LA16c-306E5.3* (forward strand), or *MTRNR2L4* (reverse strand) show no evidence of parental expression bias, while SNVs that fall uniquely within *NAA60* or *ZNF597* show almost exclusive maternal expression (Fig. 6).

Finally, careful inspection of the *TRAPPC9* locus enabled us to refine the signal of imprinting specifically to *PEG13*, which lies intronic within *TRAPPC9*. Here, we observed a cluster of SNVs located in the center of the annotated *TRAPPC9* locus showing almost exclusive paternal expression, while SNVs located elsewhere in *TRAPPC9* showed equal expression from the maternal and paternal alleles (Additional file 12). Although the gene annotations we used (Gencode v16) includes multiple isoforms of *TRAPPC9*, none included exons that corresponded to the cluster of paternally expressed SNVs within *TRAPPC9*. Instead, the use of Refseq gene annotations included the 5.6-kb transcript *PEG13* (*paternally expressed gene 13*) that, like *TRAPPC9*, is expressed from the negative strand and coincides perfectly with this cluster of paternally expressed SNVs that lie intronic within *TRAPPC9*. Thus, careful curation of this locus revealed that the imprinted signal we observed in the blood comes solely from *PEG13* and that the larger *TRAPPC9* gene is not imprinted in the cell types we studied. Thus, our observations in LCLs and blood are consistent with previous studies made in the human brain [31].

### Genome-wide scan for imprinting outside of known gene annotations

**Fig. 7:**
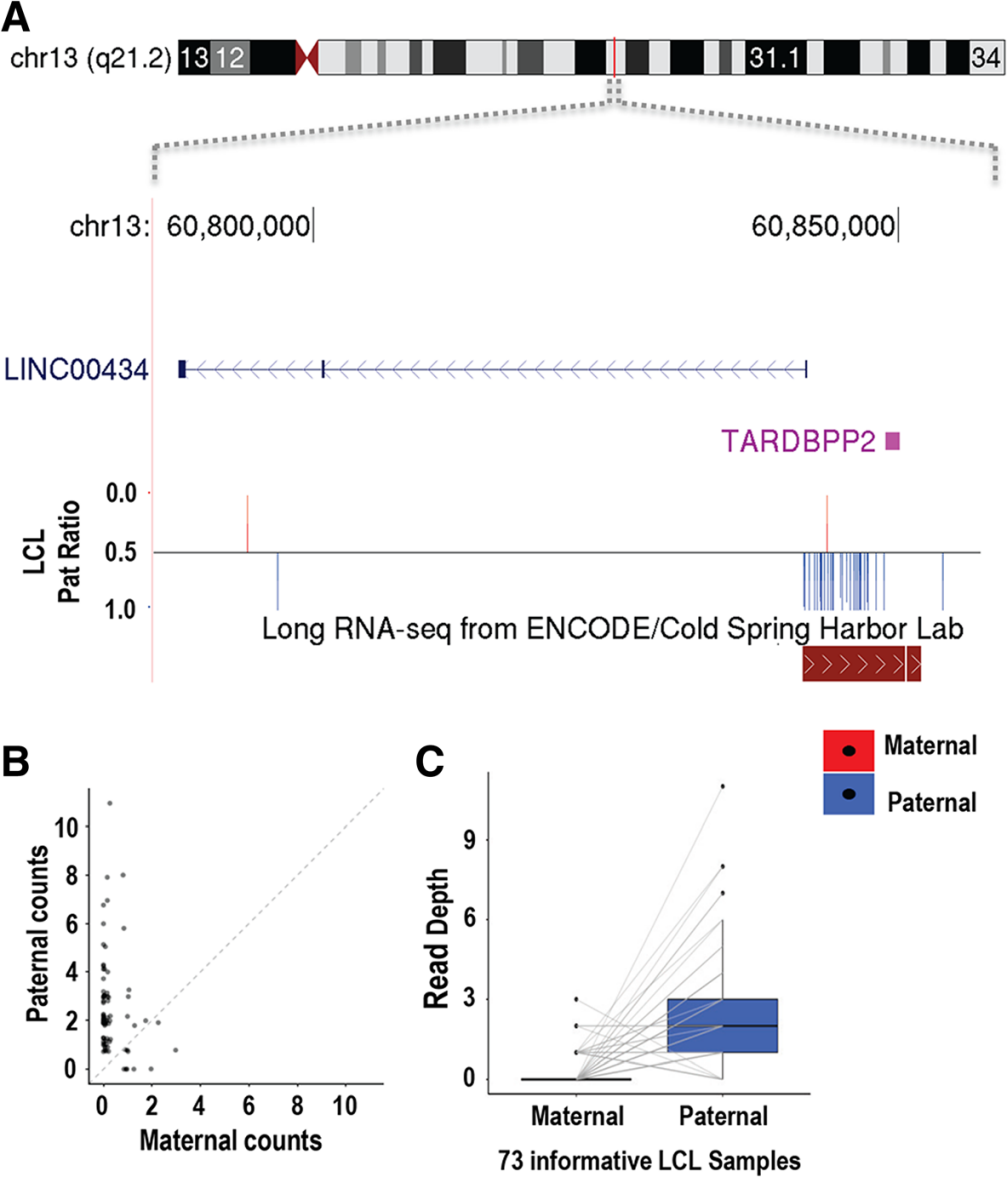
**a**–**c** A putative imprinted lncRNA at 13q21.2. Using a sliding window analysis to interrogate the genome independent of gene annotations, we identified a cluster of 35 SNVs located in 13q21.2 (chr13:60,841,936–60,848,791, hg19) that showed a strong paternal expression bias. The putative transcript containing these SNVs is located on the forward strand and apparently shares a bidirectional promoter with the non-coding RNA *LINC00434*. This SNV cluster overlaps a putative anonymous transcript identified in LCLs by the ENCODE Project

In order to search for novel signatures of imprinting outside of current gene annotations, we utilized a sliding window approach to systematically analyze the entire genome in an unbiased fashion. We chose a window size of 25 kb as this was close to the median transcript length, with a 5-kb incremental slide. At each position, we aggregated maternal and paternal read counts for all available heterozygous SNVs within the 25-kb window and calculated the WSR test statistics (Additional file 13). Using this approach, as expected, we identified significant associations at nearly all imprinted genes found using our gene-centric approach. In several cases (e.g., *ZNF331* and *ZDBF2*), significant signals of imprinted expression were observed downstream of annotated genes, which might represent transcriptional read-through beyond annotated 3′ boundaries (Additional file 14). However, we also identified a significant signal of expression outside of known gene annotations on 13q21.1 in the LCL population. Here, a cluster of 35 informative SNVs spread over ~ 8 kb showed a strong paternal bias, with 87% of reads supporting transcription from the paternal allele in 73 informative samples. We propose that this represents a maternally imprinted transcript transcribed from the forward strand that apparently shares a bidirectional promoter with *LINC00434* (Fig. 7). In support of this, data from the ENCODE Project in cell line GM12878 indicates the presence of an anonymous transcript at this position that is consistent in size and strand with our observations. There was no significant expression from this locus detected in the whole blood. Interestingly, a previous study [23] of DNA methylation in oocytes reported that the bidirectional promoter of *LINC00434* has a profile consistent with maternal-specific methylation. Additionally, Zink et al. reported *TARDBPP2* within this locus as a putatively imprinted transcript with paternal expression bias [19].

## Discussion

Here, we report a detailed survey of imprinted gene expression in two human tissues. We used a robust pipeline, incorporating the latest methods for allele-specific expression analysis, including rigorous removal of reads with potential mapping bias. The availability of phased genotype information from whole-genome sequencing of trios enables the assignment of expression levels from the two parental alleles at > 2.8 million transcribed SNVs, providing a direct approach to assess imprinting genome-wide and thereby allowing us to detect subtle imprinting effects, including genes with incomplete imprinting.

Further, we developed a robust statistical framework to account for population heterogeneity of imprinting. While many previous studies have called events at the level of individual samples and variants, we studied nearly 300 independent trios and employed two complementary statistical tests that considered aggregated read counts at the gene level across the whole population. The paired WSR is a non-parametric test that has the advantage of a low false-positive rate, but with reduced power at small sample size and low expression (Additional file 2). In contrast, SB uses the zero-inflated negative binomial distribution to fit the data, well-suited for zero-inflated count data such as RNA-Seq, providing increased power for genes with low expression. These approaches have the advantage of assessing the differences between paternal and maternal RNA-Seq counts at multiple heterozygous loci across all individuals simultaneously, thus providing both increased robustness and power to resolve subtle biases in expression from the two parental alleles, when compared to the study of single data points.

In a recent work by Zink et al., here, the authors utilized a different statistical analysis which uses a logistic regression framework to estimate PofO effect by modeling log odds ratio of reference and alternate read counts per SNV. The *p* value of the top SNV, after multiple testing correction, is then assigned to the gene. A major difference with our study is the use of ref./alt ratios per SNV, instead of aggregated counts over all SNVs per UGF, as the basis for the statistical test. The number of informative samples and zero-inflation is important factors which are captured in our study by using two different tests, WSR and SB (Additional file 2). Aggregation of counts over multiple SNVs resolves to a certain extent the sparsity issue in our study, which may be negligible when sample size is large as is the case in Zink et al. In addition, genes with fewer SNVs will show a stronger PofO effect because of multiple testing correction at the gene level which is non-existent in our study due to aggregation.

Consistent with prior studies, we found that utilizing aggregated read counts across all heterozygous sites per gene in each individual, including intronic reads and SNVs covered by only a single read, gave the most power in our analysis [11, 15]. Finally, we filtered putative imprinted transcripts to remove false signals caused by reference bias, before manually curating each locus to resolve signals from overlapping and antisense transcripts. Importantly, curation to remove reference bias was an important step to avoid false-positive imprinting signals: despite the fact that we masked non-unique genomic regions and applied stringent filtering to remove reads with ambiguous mapping, we still identified several genes with significant signals of parental expression bias which were attributable to reads mapping preferentially to the reference sequence (as assessed by statistical comparison of coverage of the reference and alternative alleles) (Additional file 7).

Overall, this pipeline led to the identification of 45 imprinted genes and one imprinted unannotated transcript in 13q21.2. Of the imprinted genes identified, two notable examples are *PER3* and *IGF2BP3*. *PER3* [Period, *Drosophila*, homolog of 3; OMIM# 603427] is a member of the Period family of genes and is expressed in a circadian pattern in multiple tissues [32]. *PER3* is one of the several genes that regulate circadian rhythms and has been linked to seasonal affective disorder by both human and mouse studies [33, 34]. *IGF2BP3* [insulin-like growth factor 2 mRNA-binding protein 3; OMIM# 608259] binds to the 5′ UTR of the imprinted gene *IGF2*, suggesting it has a role in the regulation of *IGF2* production and is expressed ubiquitously across fetal and adult tissues [35, 36]. While previous reports have shown that *IGF2BP3* is biallelically expressed, we identify a slight bias for increased expression from the paternal allele (LCL paternal ratio of 0.54). This may point at a coordinated PofO-based regulation of *IGF2* signaling cascade. Notably, a maternally methylated CpG island associated with *RPS2P32* gene lies ~ 22 kb upstream of *IGF2BP3* [25].

Classical studies of imprinting typically define imprinted genes as showing mono-allelic expression from just one of the two parental alleles. However, recent studies in mice have identified examples of incomplete, or non-canonical, imprinting [37]—such genes are biallelically expressed, but show a significant allelic bias, such that the two parental alleles are expressed at different levels. Our study also finds multiple examples of incomplete imprinting in the human genome, and we report nine imprinted genes that each shows consistent two- to threefold higher expression from the paternal allele. In several cases, these incompletely imprinted genes occur in close proximity to known imprinted genes that show mono-allelic expression, consistent with the known clustering of imprinted genes [38]. While it is possible that some of these genes with incomplete imprinting in the blood and/or LCLs might be fully imprinted (i.e., mono-allelically expressed) in other tissues, we note that none was found in a prior survey of imprinting that assayed 34 human tissues [11], making this unlikely.

Of note, we observed that some genes showed large apparent variations in paternal ratios (Fig. 2), and we found several different factors contributing to this phenomenon. In some cases, such as *PXDC1* or *PER3*, this was apparently due to stochastic variation as a result of low read depth. For example, where an individual has a single heterozygous SNV in a gene that is covered by only two RNA-Seq reads, the possible paternal expression ratios are 0, 0.5, or 1. Thus, in the case of a gene with low expression and incomplete imprinting, wide variations in the allelic ratios among different individuals will be observed as a result. In other cases, apparent variability of allelic ratios could be attributed to the fact that some genes showed isoform-specific imprinting patterns. For example, *ZNF331* has multiple different isoforms with different transcription start sites: in LCLs, those transcribed from the distal promoters show ~ 90% expression from the paternal allele, while isoforms transcribed from the most proximal promoter showed no evidence of imprinting. Thus, depending on the position of heterozygous SNVs within *ZNF331* carried by any one individual, the allelic ratio varied accordingly. Similar variability was also observed for *NAA60*, stemming from the fact that there are several overlapping annotated genes at this locus, all of which have much higher expression levels in LCLs than *NAA60*. As a result, the paternal ratio of any one SNV within *NAA60* is highly dependent upon its position within the locus. SNVs that overlap either *ZSCAN32*, *ZNF174*, or *LA16c-306E5.3* showed no evidence of parental bias, while SNVs in regions that overlap only *NAA60* or *ZNF597* showed almost exclusive maternal expression (Fig. 6).

In addition to a gene-centric approach, we also utilized a sliding window analysis to screen for imprinted transcription across the genome, independent of known transcript annotations. This identified an imprinted locus at 13q21.2, apparently corresponding to an anonymous lncRNA approximately 8 kb in length. This imprinted transcript is antisense to *LINC00434*, with the two genes apparently sharing a bidirectional promoter. Although we did not detect any expression from *LINC00434* in LCLs, given that these two genes are likely transcribed from the same promoter, we hypothesize that *LINC00434* may also be imprinted. However, this hypothesis requires formal testing in other tissues to confirm if *LINC00434* is indeed imprinted.

Given a previous report of sex-specific variations in imprinting [11], we tested whether age or gender influenced the imprinting status for any of the 46 imprinted transcripts we identified. However, we did not detect any significant effects of these two variables on parental expression bias (Additional file 15). Furthermore, as studies in mice [39, 40] have previously identified a cluster of imprinted genes on the X chromosome, and phenotypic studies in humans have led to the suggestion that genes on the human X chromosome may also be subject to imprinting [41], we specifically searched for imprinting on the X chromosome. Although this analysis utilized only female samples, and thus suffered a reduction in power compared to our analysis of the autosomes, we were unable to detect any evidence to support the presence of imprinted genes on the human X chromosome.

In order to compare our results with those published in the literature, we performed a systematic survey of genes reported as imprinted in four other population-based studies that have used RNA-Seq (Additional files 6 and 16). Overall, we observed moderate concordance among different studies, with 66 genes being reported by multiple studies and a further 159 genes reported in only a single study. Seven transcripts we identified as showing parental bias in gene expression were not reported in any of the other four studies (*EHHADH*, *IGF2BP3*, *NEK10*, *PEG13*, *PRR25*, *RP11-64J4.2*, *ZNF613*), while the majority of other singleton observations were made in the studies of Zink et al. and Babak et al. [12, 19]. While it is possible that some of these singleton observations might represent false positives, we suggest that the two major factors influencing whether a gene is reported as imprinted by a given study are likely the tissue or cell type studied, and statistical power of the study. As many genes show tissue-specific imprinting, any one study is therefore limited to observing only those genes that are imprinted in the cell type(s) being assayed. Power to discriminate significant parental expression bias is largely a function of sample size, with a large sample size allowing much more subtle PofO bias to be detected. However, power is also influenced by other factors such as the depth of RNA-Seq data obtained and the distribution of informative SNVs (which is related to both how the underlying genotypes were ascertained and sample ethnicity). These two factors largely explain why studies of multiple different tissues using GTEx data [11], and the recent study of > 11,000 individuals from the Icelandic population [19], each detected many imprinted transcripts that were not observed in other studies. Other technical factors, such as gene annotations used and experimental (e.g., the use of unstranded versus stranded RNA-Seq, or polyA+ versus ribosome-depleted RNA) and statistical methodologies (e.g., whether data is analyzed at the level of individual SNVs, or aggregated across entire transcripts, and differences in the gene annotations used), likely account for the remaining differences among studies. Such factors make it difficult to directly compare results among studies. For example, of the five studies we compared, ours was the only one to report the known imprinted gene *PEG13*, which probably results from this transcript being absent in many gene annotation sets.

However, for ten genes that were reported as imprinted in the GTEx cohort, we did not observe evidence of imprinting, despite these genes having sufficient informative SNVs to be adequately assessed in our samples (*UTS2*, *MEST*, *UBE3A*, *PLAGL1*, *CPA4*, *MAGI2*, *INPP5F_V2*, *PRSS50*, *THEGL*, *RP11-7F17.7*). We note that of these ten genes, *MEST*, *UBE3A*, *PLAGL1*, *CPA4*, *MAGI2*, and *INPP5F_V2* have all been reported as imprinted in other prior studies. While it is possible these may represent false negatives in our analysis, many apparently show tissue-specific imprinting, with normal biparental expression in the blood and LCLs, thus explaining our results [42–45]. In addition, we note that *UTS2* overlaps and is antisense to *PER3*, a gene which we identify as showing a weak paternal bias in LCLs. Given our improved methodology that utilized strand-specific RNA-Seq, we suggest that the previously reported imprinting of *UTS2* instead likely reflects paternally biased expression of *PER3*. Given the improved resolution of strand-specific over unstranded RNA-Seq data, we suggest that future expression-based studies of imprinting should utilize this approach where possible.

Our study has some limitations. Primarily, as our approach relies on measuring read depth over transcribed SNVs, we were limited to the study of genes that both contained heterozygous variants and were expressed at sufficient levels to be analyzed. Thus, genes that were not expressed at detectable levels in a sufficient number of individuals, or which lacked heterozygous variants in our samples, were not assayed. Similarly, we had a little discriminatory power to detect imprinting for genes that contained very few SNVs in our cohort or for those that were expressed at very low levels. Further, as we studied samples of peripheral blood and LCLs, we were unable to detect genes that show imprinting confined to other tissues [11]. Finally, as the LCLs we studied are immortalized cell lines, it is possible this process may have disrupted epigenetic processes such as imprinting. However, arguing against this possibility, there was both strong concordance of our results obtained in LCLs with previous studies of imprinting, and several of the imprinted genes detected in LCLs were also supported by methylation and/or RNA-Seq data from the whole blood [11].

We are aware that some previous studies have suggested that independent validation such as pyrosequencing is necessary for the robust identification of imprinted loci from RNA-Seq data [15]. This is particularly true where statistical models assume random independent sampling of reads and do not account for technical and biological variation. DeVeale et al. also suggested, based on pyrosequencing validation, several characteristic features tend to associate with genuine imprinted genes, the most prominent one of which is the presence of concordant signals of imprinting among neighboring SNVs in the same gene. In essence, this is exactly what our statistical approach does, as we chose an approach that aggregates data from multiple SNVs within each transcript annotation, thereby avoiding single SNV calls as a major source of false positives. A second feature associated with true positives highlighted by DeVeale et al. is the recurrence of a signal across biological replicates. Again, by determining the statistical significance for parental expression bias at the population level considering signal from all informative individuals, we automatically ensure recurrence across multiple individuals. In contrast, most pyrosequencing assays only assess allelic bias based on a single SNV.

## Conclusion

Given that our study assessed the imprinting status of ~ 41% of human transcripts, and identified 45 that are imprinted, our findings are broadly consistent with previous projections that have suggested that the human genome likely contains approximately 100 genes that are imprinted in somatic tissues [46].

## Methods

### Strand-specific RNA-Seq in 165 lymphoblastoid cell lines

We generated RNA-Seq data from lymphoblastoid cell lines (LCLs) for 57 CEPH (CEU), 58 Yoruba (YRI), and 50 Han Chinese (CHS) samples, all of whom were offspring of multi-generation pedigrees studied as part of The HapMap (http://hapmap.ncbi.nlm.nih.gov/) and/or 1000 Genomes (http://www.internationalgenome.org/) Projects. Samples are listed in Additional file 17.

### Genotype data processing

For 163 samples, genotype data from the complete mother/father/child trio were available, while for the two samples, genotype data for only one parent was available. We obtained 1000 Genomes and HapMap Project data from multiple releases: this included data from The 1000 Genomes Project phase 1 and phase 3 generated from low-coverage Illumina whole-genome sequencing, high coverage Complete Genomics whole-genome sequencing data, exome sequencing, Illumina Omni 2.5M SNV array data, and HapMap3 Project data genotyped on Illumina 1.6M and Affymetrix 6.0 SNV arrays. We included high-quality filtered and curated DNA genotype data from the final releases of all these resources and combined into population-specific datasets. We performed quality control on the merged data such as resolving strand inconsistencies, removing multi-allelic SNVs and indels, removing SNVs not present in the 1000 Genomes data, and converting coordinates from hg18 to hg19 where required using PLINK (versions 1.07 and 1.9) [47, 48], vcftools (version 0.1.15) [49], and Beagle Utilities.

Due to the differing genotyping approaches and resulting SNV densities available across different individuals, we performed combined imputation and phasing to increase SNV density and infer the two parental haplotypes in each offspring with Beagle 4.0 [50]. This used family pedigree information with the 1000 Genomes phase 3 reference panel downloaded from the Beagle website (http://bochet.gcc.biostat.washington.edu/beagle/1000_Genomes_phase3_v5a/). Using 493 HapMap samples from the CEU, YRI, and CHS populations, we created population-specific reference panels to improve the imputation accuracy. Since many of the samples in our target panel are also part of 1000 Genomes Project reference panel, for each population group, we created subsets of target and reference panel in such a way that there are no overlapping samples in two sets and imputed and phased each of these subsets of target panel separately. Each chromosome was divided into segments to efficiently perform imputation and phasing, and these segments were subsequently merged together to yield chromosome-wide imputed and phased genotypes. Imputed genotypes were filtered to retain only high-quality genotypes (*R*^2^ ≥ 0.95). We also removed sites with Mendelian errors in each trio, Hardy-Weinberg equilibrium *p* < 10^−4^, and retained only biallelic SNVs with minor allele frequency ≥ 5% in at least one of the three ethnicities in the cohort. This yielded ~ 3.9 million high-quality SNVs phased for parental origin.

To reduce phase switch errors introduced during phasing that would result in incorrect parental origin assignment of SNVs, we used an R script developed in-house (https://github.com/SharpLabMSSM/PofOAssignment). This method utilizes the phased genotypes generated using BEAGLE, as follows: Each offspring’s haplotype is compared with the parental haplotypes using a sliding window of 100 SNVs with 50 SNV incremental slide. Within each window, we check for perfect matches between each offspring haplotype and the four possible haplotypes within the parents. Parental origin assignments for each haplotype in the offspring are based on an unambiguous match to a single parental haplotype. This approach allows assignment of parental origin at uninformative sites where all members of the trio are heterozygous and also provides an error check for phase switching. In the case when offspring’s haplotypes do not perfectly match a parental haplotype, the genotypes in the window are set to missing. Subsequently, we then recover any such lost sites using simple rules of Mendelian inheritance to each individual SNV genotype in the trio. Thus, by using a combined approach leveraging both statistical phasing with rules of Mendelian inheritance, we are able to generate maximally informative assignment for parental origin at heterozygous SNVs, with a minimal error rate.

### Sample preparation

Lymphoblastoid cell lines were obtained from the Coriell Institute (Camden, NJ). Cells were grown in RPMI1640 media supplemented with 1 mM l-glutamine, 10% FBS, and 100u/L each of penicillin and streptomycin, according to the recommended protocols. Total RNA was extracted from frozen cell pellets (5–10 million cells) using TRIZOL, according to the manufacturer’s instructions (ThermoFisher Scientific). Strand-specific RNA-Seq libraries were prepared using NEBNext Ultra Directional RNA Library Prep Kit from Illumina. One microgram of total RNA was used as input, polyA+ selected, followed by strand synthesis was performed. Libraries were sequenced on an Illumina Hiseq 2500 instrument, with 10 samples pooled per lane, to generate 100 bp single-end reads to a median depth of ~ 16 million reads per sample.

### RNA-Seq data processing

Quality control analysis was performed on RNA-Seq reads using fastqc (version 0.11.2) (http://www.bioinformatics.babraham.ac.uk/projects/fastqc). Over-represented sequences were removed using trimmomatic (version 0.32) [51], and trimmed reads ≥ 30 bp in length were kept. Cleaned reads were mapped to the human reference genome (hg19) with Gencode v16 annotations using the STAR aligner (version 2.3.0) [52], yielding a mean of 79% uniquely mapped reads. Picard (version 1.112) (https://github.com/broadinstitute/picard) was used for intermediate BAM file processing such as add read groups and sorting and merging BAM files of the same samples. To correct for mapping errors and biases which can result in false-positive allele-specific read assignments, we used a collection of utilities in the WASP software (version 0.1) [53], resulting in the removal of a mean of 36% of reads that overlapped SNVs in each sample, for which unambiguous allelic assignment could not be made. After parental origin assignment for SNVs in each offspring, heterozygous sites were used to determine allele-specific expression. We first quantified reference and alternate RNA-Seq reads mapped at heterozygous loci using AlleleCounter (v0.2, https://github.com/secastel/allelecounter) implemented in Python [10]. Then, reference and alternate allele counts were used with PofO information to assign counts to the maternal and paternal alleles at each heterozygous site. Reads that did not uniquely map, or had base quality ≤ 10, were discarded. To further reduce the mapping errors, we applied additional filters, removing heterozygous SNVs that (i) had a mappability score < 1 (based on the “CRG GEM Alignability of 50mers with no more than 2 mismatches” track, downloaded from UCSC genome browser), (ii) overlapped CNVs with MAF ≥ 5% identified in samples from the 1000 Genomes and HapMap Projects (ftp://ftp.1000genomes.ebi.ac.uk/vol1/withdrawn/phase3/integrated_sv_map/ and common CNVs [54], (iii) segmental duplications, and (iv) simple repeats (both downloaded from “Variation and Repeats” track group of the UCSC genome browser). These filters resulted in the removal of 21% of heterozygous sites, leaving ~ 3.1 million sites for downstream analysis.

### Unstranded RNA-Seq in 131 whole blood samples

The Genome of the Netherlands (GoNL) Project [20] performed whole-genome sequencing of 250 family trios, a subset of which also had whole blood transcriptomes sequenced as part of the BBMRI-NL Biobank-based Integrative Omics Study (BIOS) [55, 56]. From these, we utilized data from 131 children with whole blood RNA-Seq data that passed all quality criteria and had genotypes concordant with those obtained by whole-genome sequencing (listed in Additional file 18). The individuals were participants from one of four biobanks: LifeLines-DEEP, The Leiden Longevity Study, Netherlands Twin Registry, and the Rotterdam Study.

#### Genotype data processing

DNA genotypes of 250 Dutch families were phased and imputed using BEAGLE [57] and IMPUTE2. An integrated phase panel was constructed using SNV genotype likelihoods from the GATK:UnifiedGenotyper as input for BEAGLE, treating all samples as unrelated. SHAPEIT2 and MVNcall19 were then used along with trio information to phase the complete set of SNVs. Each haplotype transmitted to the offspring, and therefore, allelic parental origin was then obtained from the phased haplotypes [20].

#### Sample preparation

Total RNA from the whole blood was treated using Ambion’s GLOBIN clear kit and subsequently processed for sequencing using the Illumina Truseq version 2 library preparation kit. Paired-end 50 bp reads were generated using an Illumina HiSeq 2000 instrument, pooling 10 samples per lane. Read sets per sample were generated using CASAVA, retaining only reads passing Illumina’s chastity filter for further processing. Data was generated by the Human Genotyping Facility (HugeF) of ErasmusMC (The Netherlands, see URLs). Full details are described in [55].

#### RNA-Seq data processing

Initial quality control was performed using FastQC (v0.10.1). Removal of adaptors was performed using Cutadapt (v1.1) [58]. Sickle (v1.2) [59] was used to trim low-quality ends of the reads (minimum length 25, minimum quality 20). The reads were mapped with the STAR aligner (v2.3.125) [52] to human reference genome hg19 masked at all single nucleotide variants with MAF > 0.01 in GoNL samples. Full details are described in [55]. To reduce the influence of reference bias, we utilized WASP (version 0.1) [53] to remove reads that aligned to different genomic positions after substituting the variant site. A summary of the influence of masking SNV positions in the reference and utilizing WASP to remove reads that show ambiguous mapping positions is shown in Additional file 19.

To obtain the parent-of-origin allelic counts, we first computed RNA-Seq reference and alternative counts using the GATK (v3.6-0-g89b7209) ASEReadCounter tool [60]. A script was then used to re-label the reference and alternative counts with parental origin based on the transmitted allele, leaving ~ 0.9 million heterozygous sites with paternal and maternal read counts for downstream analysis. A summary of the complete analytical pipeline is shown in Additional file 20.

#### Statistical analysis to identify imprinted expression

Since overlapping genes are common in the eukaryotic genome [61], care must be taken when assigning reads to specific transcripts. To avoid misassignment of reads at SNVs located within the overlapping transcripts, we compiled all genes from Gencode annotations into a model where we consider the overlapping regions of different genes as a separate unit, termed as “unique gene fragments” (UGFs) (Additional file 21). The resulting gene models comprised 79,452 UGFs and were used for assigning each heterozygous SNV to specific genes.

To maximize the statistical power for detecting PofO-biased expression, we summed the read counts for all SNVs within each UGF. We calculated the paternal allelic ratio (defined as the fraction of reads derived from the paternally inherited allele) for each individual using aggregated read counts across all informative SNVs within each UGF. We used the paternal allelic ratio of each informative individual to calculate the mean paternal ratio per UGF.

To formally test for parental bias in the expression of UGFs, we utilized two complementary statistical approaches. We chose (i) a frequentist non-parametric approach, the paired Wilcoxon signed-rank (WSR) test and (ii) an empirical Bayes approach *ShrinkBayes* [62]. ShrinkBayes computes a Bayesian false discovery rate (BFDR), and we applied Benjamini-Hochberg false discovery rate (FDR) correction to the results of the WSR test, considering those UGFs with FDR *q* < 0.1 (10% FDR) as showing significant evidence of imprinting. In each cohort, we only considered results for those genes in which at least 10% of individuals had ≥ 1 read informative for parental origin. Based on the results of these two tests, we classified predicted imprinted genes into those with high confidence (identified as significant by both tests) and low confidence (significant by one of the two tests). WSR test is a paired difference non-parametric test. It assigns ranks to the paternal/maternal differences with *H*_0_: mean difference in pairs is symmetric around 0. The test is robust against outliers and has no distributional assumption. ShrinkBayes is an advanced statistical method specifically designed to handle zero-inflated count data allowing multi-parameter inference and modeling of random effects in a Bayesian setting. It relies on INLA [63] for the parameter estimation per gene while borrowing information across genes by empirical Bayes-type shrinkage of parameters. It allows a spike-and-slab prior for the parameter of interest (*patmat: mean difference in pairs*) to test *H*_0_. Per UGF, we use a simple model with a single predictor parameter for imprinting (patmat) and a random effect parameter (indiv) to account for within-individual variability.

$$ y\sim 1+\mathrm{patmat}+f\left(\mathrm{indiv}\right) $$

To assess the performance of the test procedures ShrinkBayes and WSR, we developed a simulation scheme. ShrinkBayes is superior to WSR in terms of statistical power (Additional files 1 and 2) at a cost of increased computational resources. Using the two tests together reduces the false-positive rate (Additional file 1), which motivates our definition of high-confidence genes.

Following statistical testing, we manually curated the UGF level results based on visual inspection of data plots, considering both gene annotations and strand-specific data in LCLs. Here, we removed redundancies, and in the case of overlapping transcripts, assigned imprinted expression to the correct gene. At several loci where we detected imprinted expression, gene annotations included transcripts with anonymous clone IDs. An example of this is the *L3MBTL1*/*SGK2* locus on chromosome 20. Here, Gencode annotations include a transcript *RP1-138B7.5*, which is almost identical to an isoform of *SGK2*. In such cases, even though the transcript *RP1-138B7.5* was included in our initial list of significant imprinted genes, to avoid artificially inflating the number of imprinted transcripts we report, where these anonymous clone IDs likely corresponded to other annotated genes, we did not report them in our final curated list (Tables 1 and 2). Furthermore, although we filtered reads for potential mapping bias using WASP, we performed an additional check of UGF-level data for reference bias. We aggregated reference and alternate allele read counts at the UGF level and applied a two-sided WSR test to check whether the distribution of reference and alternate read counts was significantly different after multiple testing corrections (5% FDR), removing genes that showed significant reference bias.

#### Chromosome X analysis

To assess if any genes on the X chromosome were expressed in a PofO-specific manner, we conducted analyses of female samples in both LCLs (*n* = 68) and WB (*n* = 77) samples, taking into account the potential confounder of unequal X chromosome inactivation ratios (XCIR). In each female, we used maternal and paternal read counts data for all X-linked genes containing heterozygous variants to calculate the XCIR:

$$ \mathrm{XCIR}=\frac{\sum_{i=1}^m\mathrm{patCoun}{\mathrm{t}}_i\ }{\sum_{i=1}^m\mathrm{patCoun}{\mathrm{t}}_i+{\sum}_{i=1}^m\mathrm{matCoun}{\mathrm{t}}_i\ } $$

where *m* is the number of genes. We excluded females with skewed XCIR (ratios either < 0.2 or > 0.8), which left 45 females in the LCL and 67 in WB cohort. In the remaining females, we adjusted the maternal and paternal read counts of X-linked genes using the XCIR measured in each individual. Finally, we applied the paired Wilcoxon signed-rank test using the XCIR-weighted maternal and paternal read counts of X-linked genes.

## Additional files

Additional file 1: Power estimates for ShrinkBayes and the paired Wilcoxon signed-rank test on the number of genes (L) and samples (R). To assess the performance of the test procedures SB and WSR test, we developed a simulation scheme with the number of genes and individuals as parameters. RNA Seq data is simulated using ssizeRNA R package (v1.2.8) capable of simulating count data for two-group differential gene expression analysis with additional parameters for fold change, dispersion, and size (expression level). We model imprinting in an individual with expression fold change in one of the parents. We labeled the groups as paternal and maternal and assigned a factor of 2 fold change to one group and thus simulating imprinting. For a better approximation of the real data, we generated different expression levels from low to high with different proportions and fixed dispersion to 0.4. We use count level categories {2,10,20,50,100,500} with corresponding proportions of genes {0.5,0.2,0.1,0.1,0.07,0.03} having those count levels. Note that for the sake of approximation, we used fixed values 138 and 24,597 for the number of individuals and genes, respectively, corresponding roughly to the reported aggregated GoNL data in the manuscript. The expression levels, dispersion, and fold change are fixed for all simulations. We also fix the number of imprinted genes to 1% of the total number of genes. The imprinting is simulated by assigning a factor of twofold change to the paternal label. The same expression level and proportions are used for the 99% non-imprinted gene but with fold change = 1. (TIF 879 kb)

Additional file 2: Putative imprinted UGFs identified by ShrinkBayes and/or the paired Wilcoxon signed-rank test as a function of underlying sample size (L) and mean expression (R). Each box plot shows transcript fragments with significant evidence of imprinting that were (left) high confidence (identified by both SB and WSR tests), (middle) identified by SB only, and (right) identified by WSR only (FDR *q* < 0.1). Each UGF was subject to manual curation of raw data and classified as a true positive (TP, blue) or false positive (FP, red). The LC category (SB and WSR) shows a clear difference in the test performance: SB is more sensitive at reduced sample size and expression, although WSR still identified many signals that are missed by SB. We conclude that signals of imprinting identified by both tests are the most robust, while each test is able to detect additional signals, albeit with a higher false-positive rate. (TIF 711 kb)

Additional file 3: 78 significant unique gene fragments. All UGFs with FDR *q* < 0.1, prior to manual curation. (XLSX 113 kb)

Additional file 4: All unique gene fragments tested in this analysis. Data for all UGFs in the genome. (XLSX 19820 kb)

Additional file 5: Overlap of identified genes in two tissues and two statistical methods. (A) 51% of genes identified as imprinted genes were concordant in both LCLs and whole blood. (B) In LCLs, 89% of the genes that were scored as imprinted were detected by both Wilcoxon signed-prank test and ShrinkBayes. (C) In the whole blood, 63% of the genes that were scored as imprinted were detected by both Wilcoxon signed-rank test and ShrinkBayes. (TIF 423 kb)

Additional file 6: Comparison of LCL/WB imprinting results with previous studies. We list all genes identified as imprinted either in our dataset, as well as those reported as imprinted in recent studies that used RNAseq data in either the GTEx cohort or the Icelandic population. (XLSX 41 kb)

Additional file 7: Reference bias can cause false-positive signals of imprinting. A screen for imprinted genes on the X chromosome identified two putative imprinted transcripts, which were both found to be false-positive associations due to reference bias. (A) UCSC Genome Browser view showing a single informative SNV within RNA28S5, a pseudogene at Xq22.3. (B) Scatter plot and (C) table of reference and alternate read counts in six female LCLs heterozygous for rs190908473 shows that > 98% of reads overlapping this SNP match the reference genome, indicating the putative maternal expression bias is caused by a read mapping bias. (D) ARSD showed a putative maternal expression bias in samples of whole blood. (E) However, informative RNA Seq reads from ARSD showed a strong mapping bias to the alternative (non-reference) allele, indicating this as a false-positive association. (TIF 588 kb)

Additional file 8: Results of clonality analysis in LCLs. Reanalysis of unique gene fragments with FDR *q* < 0.1 using only 45 non-clonal female LCLs without skewed X chromosome inactivation ratios. (XLSX 20 kb)

Additional file 9: Methylation profiles at imprinted loci in whole blood, LCLs, and samples with genome-wide maternal or paternal uniparental disomy (matUPD and patUPD). To assess whether there is a loss of methylation at imprinted loci in LCLs, we gathered available Illumina 450 k methylation data from whole blood (1419 samples taken from six published studies on GEO) [67], HapMap LCLs (133 samples from GEO dataset GSE39672), and whole blood from individuals with genome-wide uniparental disomy (UPD) (taken from GEO dataset GSE52576). The plot shows mean DNA methylation levels at 48 differentially methylated regions associated with imprinted genes that show parental-specific methylation [24]. LCLs and whole blood show very similar methylation profiles at all imprinted DMRs. In contrast for the six paternally methylated DMRs (left side), methylation in maternal UPD samples is much lower than either blood or LCLs. Similarly, for the 42 maternally methylated DMRs (right side), samples with paternal UPD show much lower methylation than either blood or LCLs. Thus, we conclude that there is no evidence for loss of imprinting in LCLs and that methylation at imprinted DMRs is generally very similar in the blood and LCLs. (TIF 794 kb)

Additional file 10: Location of closest imprinted differentially methylated region from two published studies to each imprinted gene identified in our dataset. (XLSX 17 kb)

Additional file 11: Example of strand-specific data showing paternal expression bias at *PER3/RP3-467L1.4* locus. *PER3* and *RP3-467L1.4* are two overlapping genes transcribed from opposite strands. *PER3* shows incomplete imprinting, whereas *RP3-467L1* shows stronger paternal bias in LCLs (paternal ratios = 0.61 and 0.81, respectively). (TIF 2840 kb)

Additional file 12: Complex imprinting at *HM13* and the *TRAPPC9-PEG13* locus. (A) The longest isoform of *HM13* shows paternal expression bias, while shorter isoforms are apparently biallelically expressed. (B) *PEG13* shows exclusive paternal expression, while *TRAPCC9* is biallelic. (TIF 1281 kb)

Additional file 13: All 25 kb windows with significant *p* values from sliding window analysis. (XLSX 478 kb)

Additional file 14: Examples of significant signals of imprinting that extend beyond the annotated boundaries of genes. Possible transcriptional read-through beyond gene annotations at *ZNF331*, *ZDBF2*, and *GNAS-AS1* locus. (TIF 807 kb)

Additional file 15: Analysis of potential age and gender effects on imprinting. (PDF 111 kb)

Additional file 16: Venn diagram showing an overlap of imprinted genes reported by five recent studies that utilized RNA-Seq. (TIF 657 kb)

Additional file 17: 165 LCLs used for RNA-Seq analysis and their parents. (XLSX 20 kb)

Additional file 18: 131 whole blood samples used for RNA-Seq analysis. (XLSX 12 kb)

Additional file 19: The effect of masking SNV positions and utilizing WASP on reference genome mapping bias. Utilizing an unmasked reference genome, the median alternate ratio was 0.458. This increased to 0.483 after masking common SNV positions (SNVs with MAF > 0.01 were replaced by “N”) and further increased to the theoretical expectation of 0.5 after utilizing WASP to remove reads with ambiguous mapping positions. (TIF 312 kb)

Additional file 20: A summary of the analytical pipeline used for identifying parental bias in gene expression in whole blood samples. (TIF 408 kb)

Additional file 21: Definition of unique gene fragments (UGFs). In order to avoid misassignment of reads at SNVs located within overlapping transcripts during our gene-centric analysis, we compiled all genes from Gencode annotations into a gene model where we consider overlapping regions of different genes as a separate gene. We termed these annotations unique gene fragments (UGFs). Statistical testing on each UGF was performed, and after all significant associations were compiled (Additional files 3 and 4), we manually curated each signal and removed redundant annotations, reporting a final list of 45 imprinted genes (Tables 1 and 2). (TIF 210 kb)

Additional file 22: Members of the *Biobank-based Integrative Omics Study* (BIOS) Consortium. (PDF 68 kb)

Additional file 23: Members of the *Genome of the Netherlands* (GoNL) Consortium. (PDF 43 kb)

## Data Availability

The raw and processed RNA-Seq data for 165 LCL samples have been deposited in the NCBI GEO database under accession number GSE92521 [64]. The 131 WB STAR-aligned BAM files (freeze 2) are submitted to the European Genome-phenome Archive (EGA) under study EGAS00001001077 and dataset accession number EGAD00001003937 [65]. The phased/imputed SNV data are part of the Genome of the Netherlands (GoNL) Project with EGA accession number EGAS00001000644 [66].

## Abbreviations

GoNL: Genome of the Netherlands
LCL: Lymphoblastoid cell line
PofO: Parent-of-origin
SB: ShrinkBayes
UGF: Unique gene fragments
WB: Whole blood
WSR: Paired Wilcoxon signed-rank test
