# Supplementary figures and images for: RNA-Seq in 296 phased trios provides a high-resolution map of genomic imprinting
Source: BMC Biol. 2019 Jun 24;17:50. doi: 10.1186/s12915-019-0674-0 (PMC6589892; doi:10.1186/s12915-019-0674-0)

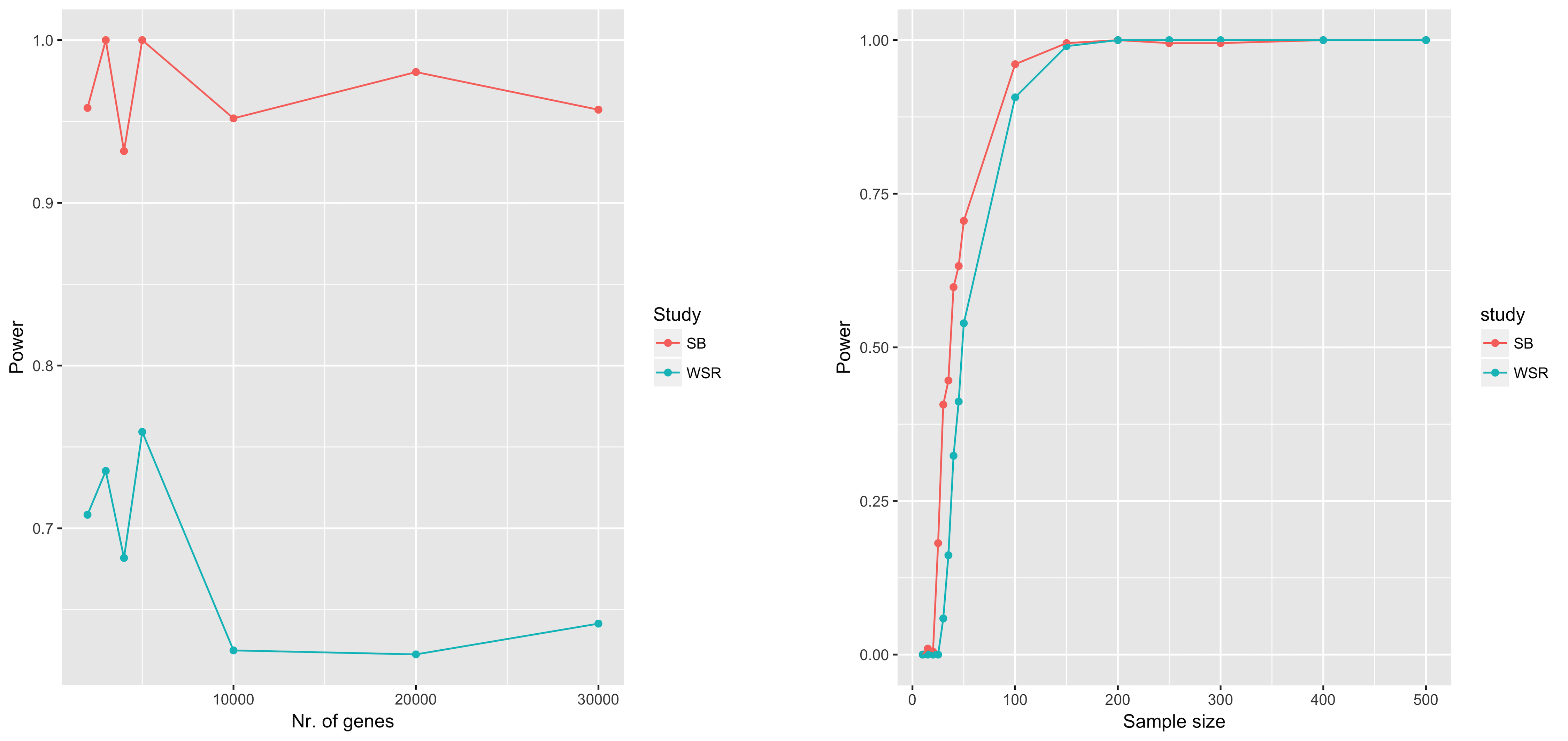

Supplement: Supplementary file 1 — Power estimates for ShrinkBayes and the paired Wilcoxon signed-rank test on the number of genes (L) and samples (R). To assess the performance of the test procedures SB and WSR test, we developed a simulation scheme with the number of genes and individuals as parameters. RNA Seq data is simulated using ssizeRNA R package (v1.2.8) capable of simulating count data for two-group differential gene expression analysis with additional parameters for fold change, dispersion, and size (expression level). We model imprinting in an individual with expression fold change in one of the parents. We labeled the groups as paternal and maternal and assigned a factor of 2 fold change to one group and thus simulating imprinting. For a better approximation of the real data, we generated different expression levels from low to high with different proportions and fixed dispersion to 0.4. We use count level categories {2,10,20,50,100,500} with corresponding proportions of genes {0.5,0.2,0.1,0.1,0.07,0.03} having those count levels. Note that for the sake of approximation, we used fixed values 138 and 24,597 for the number of individuals and genes, respectively, corresponding roughly to the reported aggregated GoNL data in the manuscript. The expression levels, dispersion, and fold change are fixed for all simulations. We also fix the number of imprinted genes to 1% of the total number of genes. The imprinting is simulated by assigning a factor of twofold change to the paternal label. The same expression level and proportions are used for the 99% non-imprinted gene but with fold change = 1. (TIF 879 kb) [file 12915_2019_674_MOESM1_ESM.tif]

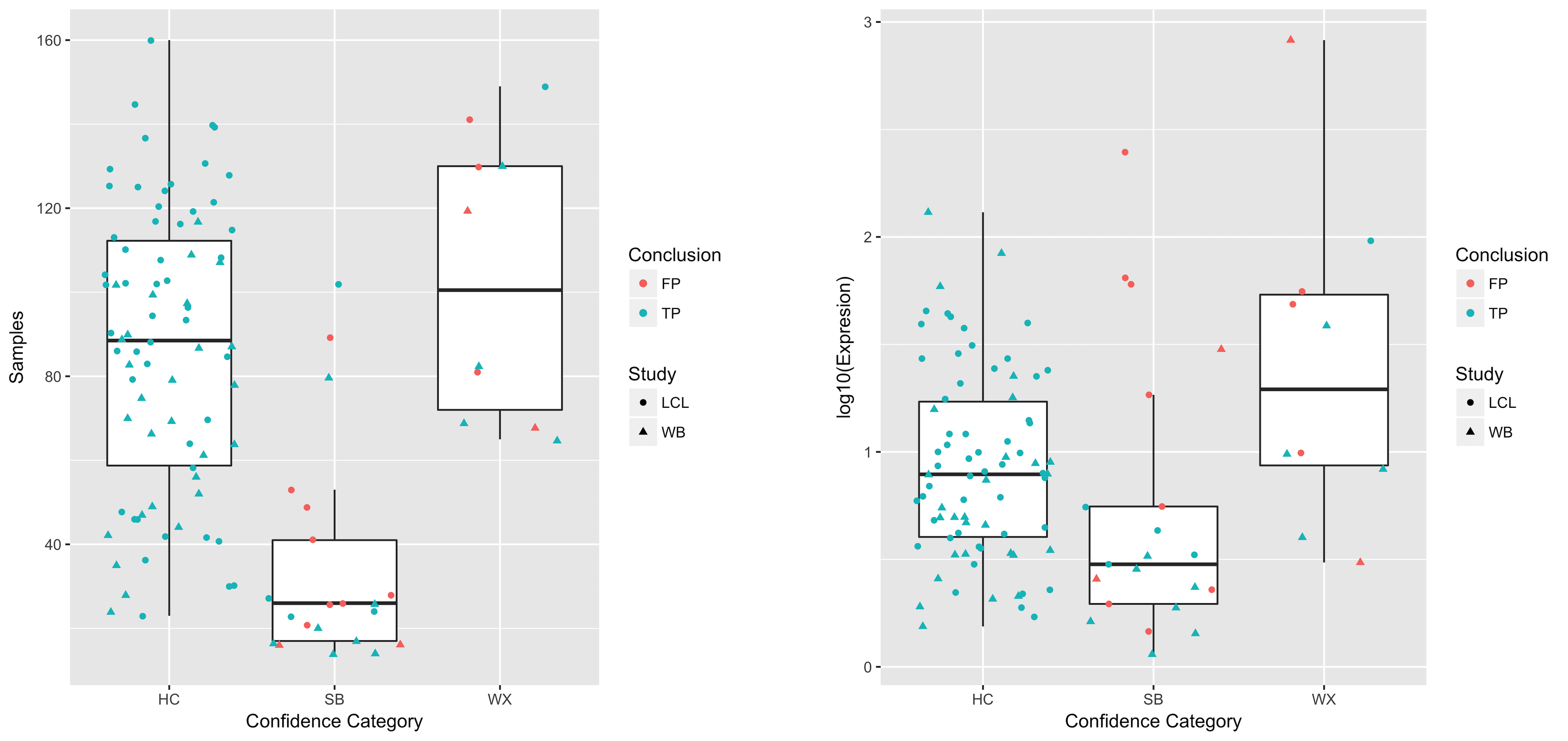

Supplement: Supplementary file 2 — Putative imprinted UGFs identified by ShrinkBayes and/or the paired Wilcoxon signed-rank test as a function of underlying sample size (L) and mean expression (R). Each box plot shows transcript fragments with significant evidence of imprinting that were (left) high confidence (identified by both SB and WSR tests), (middle) identified by SB only, and (right) identified by WSR only (FDR q < 0.1). Each UGF was subject to manual curation of raw data and classified as a true positive (TP, blue) or false positive (FP, red). The LC category (SB and WSR) shows a clear difference in the test performance: SB is more sensitive at reduced sample size and expression, although WSR still identified many signals that are missed by SB. We conclude that signals of imprinting identified by both tests are the most robust, while each test is able to detect additional signals, albeit with a higher false-positive rate. (TIF 711 kb) [file 12915_2019_674_MOESM2_ESM.tif]

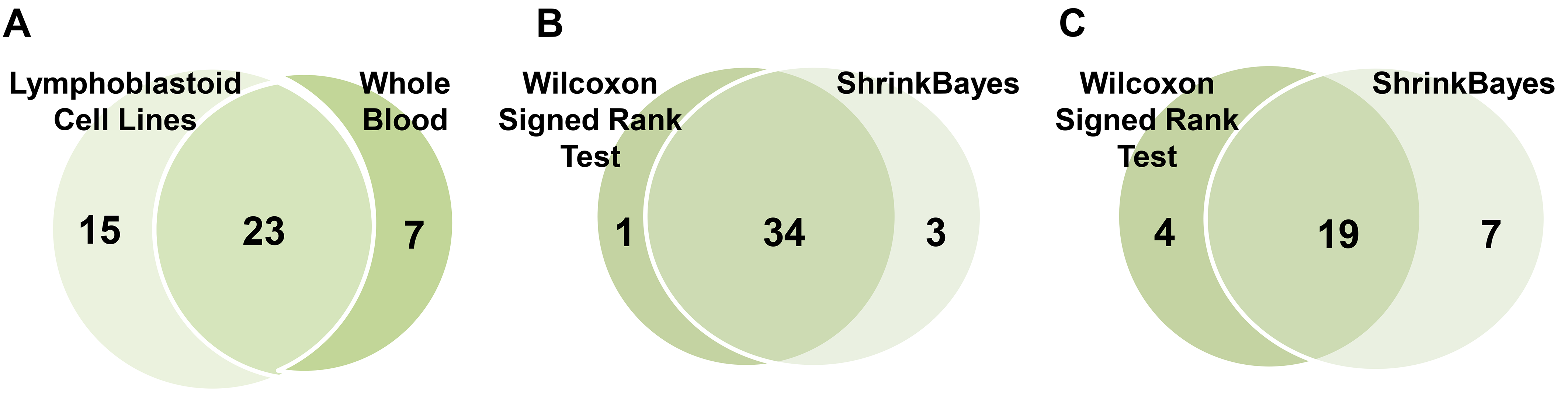

Supplement: Supplementary file 5 — Overlap of identified genes in two tissues and two statistical methods. (A) 51% of genes identified as imprinted genes were concordant in both LCLs and whole blood. (B) In LCLs, 89% of the genes that were scored as imprinted were detected by both Wilcoxon signed-prank test and ShrinkBayes. (C) In the whole blood, 63% of the genes that were scored as imprinted were detected by both Wilcoxon signed-rank test and ShrinkBayes. (TIF 423 kb) [file 12915_2019_674_MOESM5_ESM.tif]

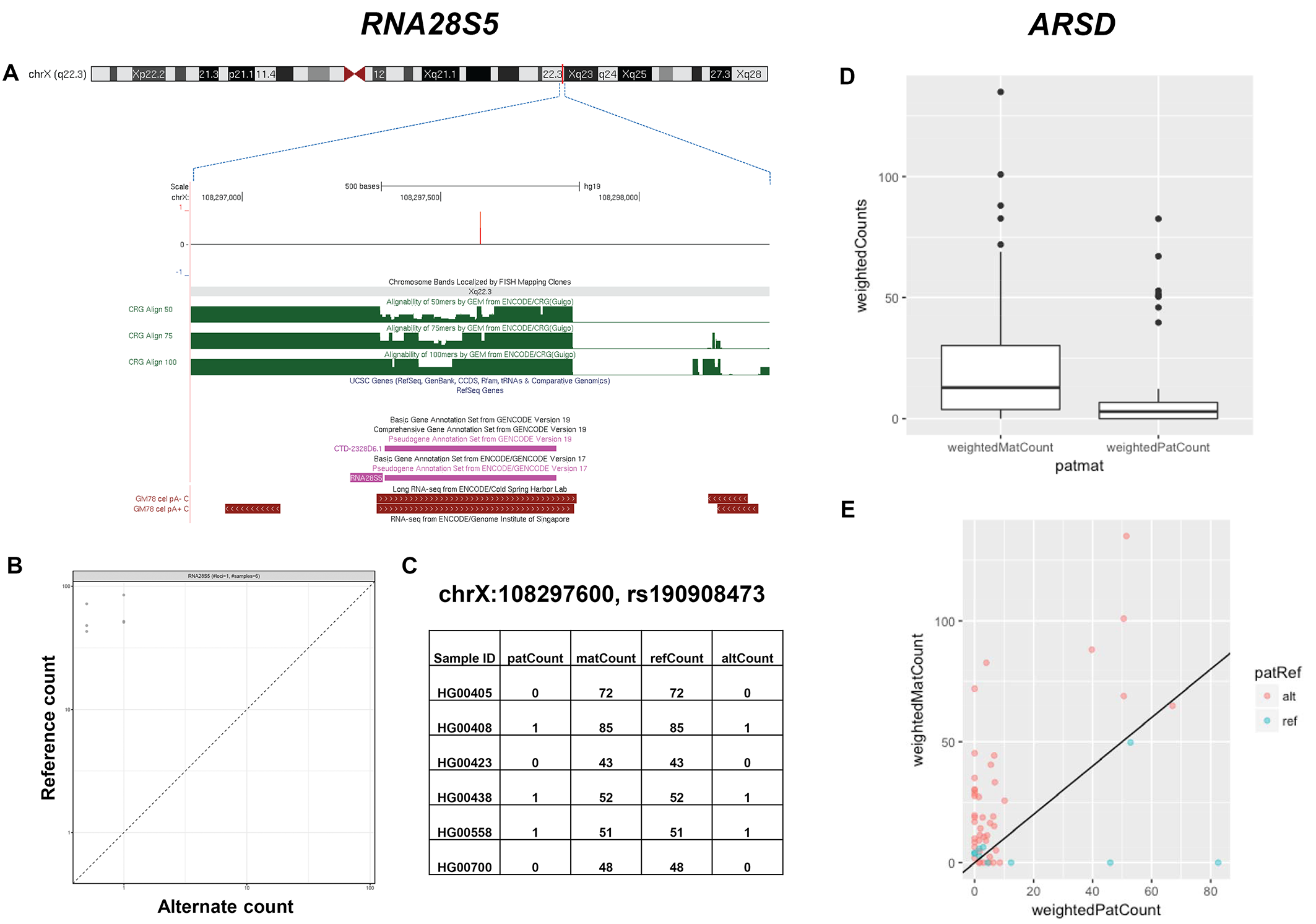

Supplement: Supplementary file 7 — Reference bias can cause false-positive signals of imprinting. A screen for imprinted genes on the X chromosome identified two putative imprinted transcripts, which were both found to be false-positive associations due to reference bias. (A) UCSC Genome Browser view showing a single informative SNV within RNA28S5, a pseudogene at Xq22.3. (B) Scatter plot and (C) table of reference and alternate read counts in six female LCLs heterozygous for rs190908473 shows that > 98% of reads overlapping this SNP match the reference genome, indicating the putative maternal expression bias is caused by a read mapping bias. (D) ARSD showed a putative maternal expression bias in samples of whole blood. (E) However, informative RNA Seq reads from ARSD showed a strong mapping bias to the alternative (non-reference) allele, indicating this as a false-positive association. (TIF 588 kb) [file 12915_2019_674_MOESM7_ESM.tif]

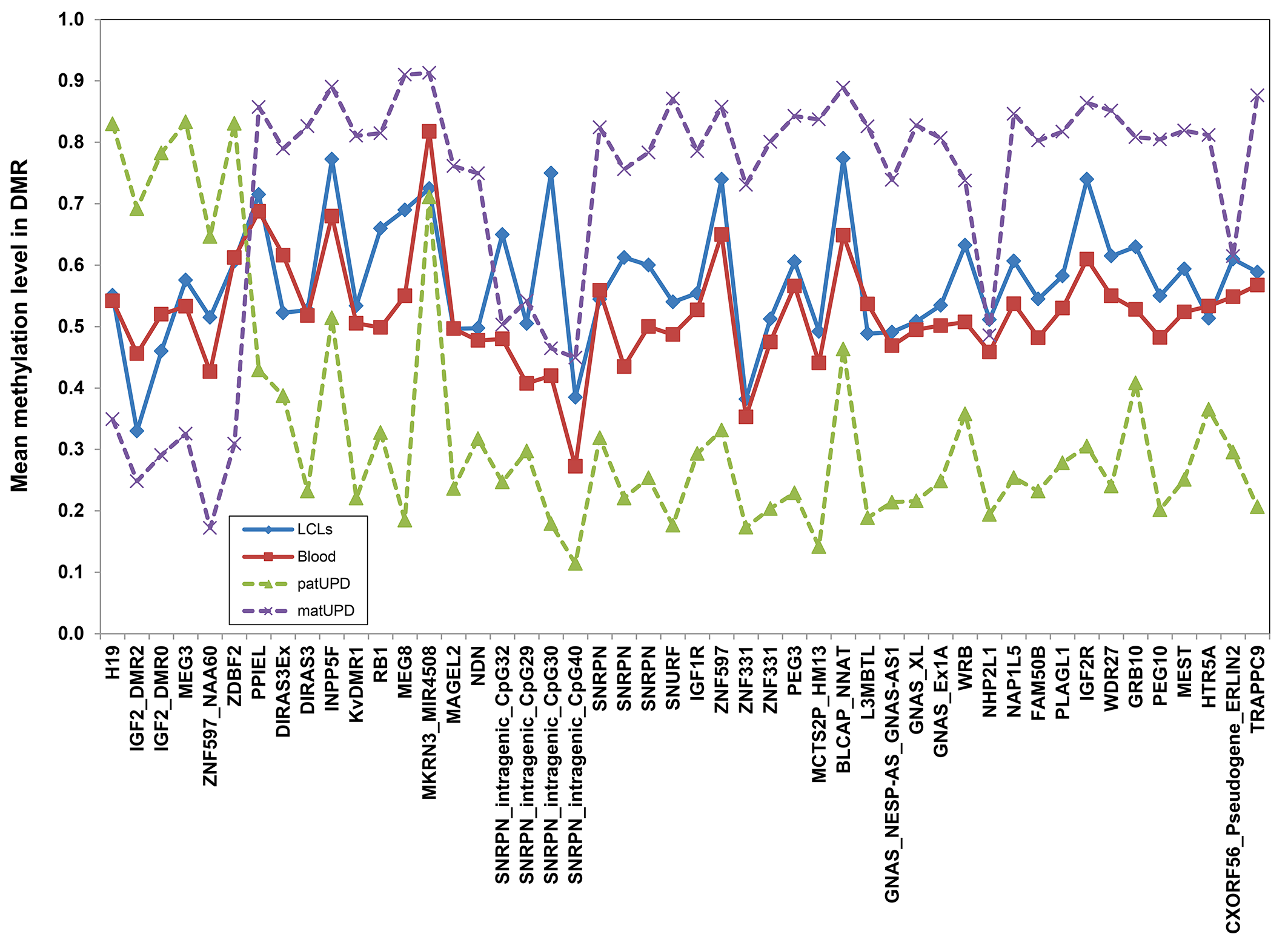

Supplement: Supplementary file 9 — Methylation profiles at imprinted loci in whole blood, LCLs, and samples with genome-wide maternal or paternal uniparental disomy (matUPD and patUPD). To assess whether there is a loss of methylation at imprinted loci in LCLs, we gathered available Illumina 450 k methylation data from whole blood (1419 samples taken from six published studies on GEO) [67], HapMap LCLs (133 samples from GEO dataset GSE39672), and whole blood from individuals with genome-wide uniparental disomy (UPD) (taken from GEO dataset GSE52576). The plot shows mean DNA methylation levels at 48 differentially methylated regions associated with imprinted genes that show parental-specific methylation [24]. LCLs and whole blood show very similar methylation profiles at all imprinted DMRs. In contrast for the six paternally methylated DMRs (left side), methylation in maternal UPD samples is much lower than either blood or LCLs. Similarly, for the 42 maternally methylated DMRs (right side), samples with paternal UPD show much lower methylation than either blood or LCLs. Thus, we conclude that there is no evidence for loss of imprinting in LCLs and that methylation at imprinted DMRs is generally very similar in the blood and LCLs. (TIF 794 kb) [file 12915_2019_674_MOESM9_ESM.tif]

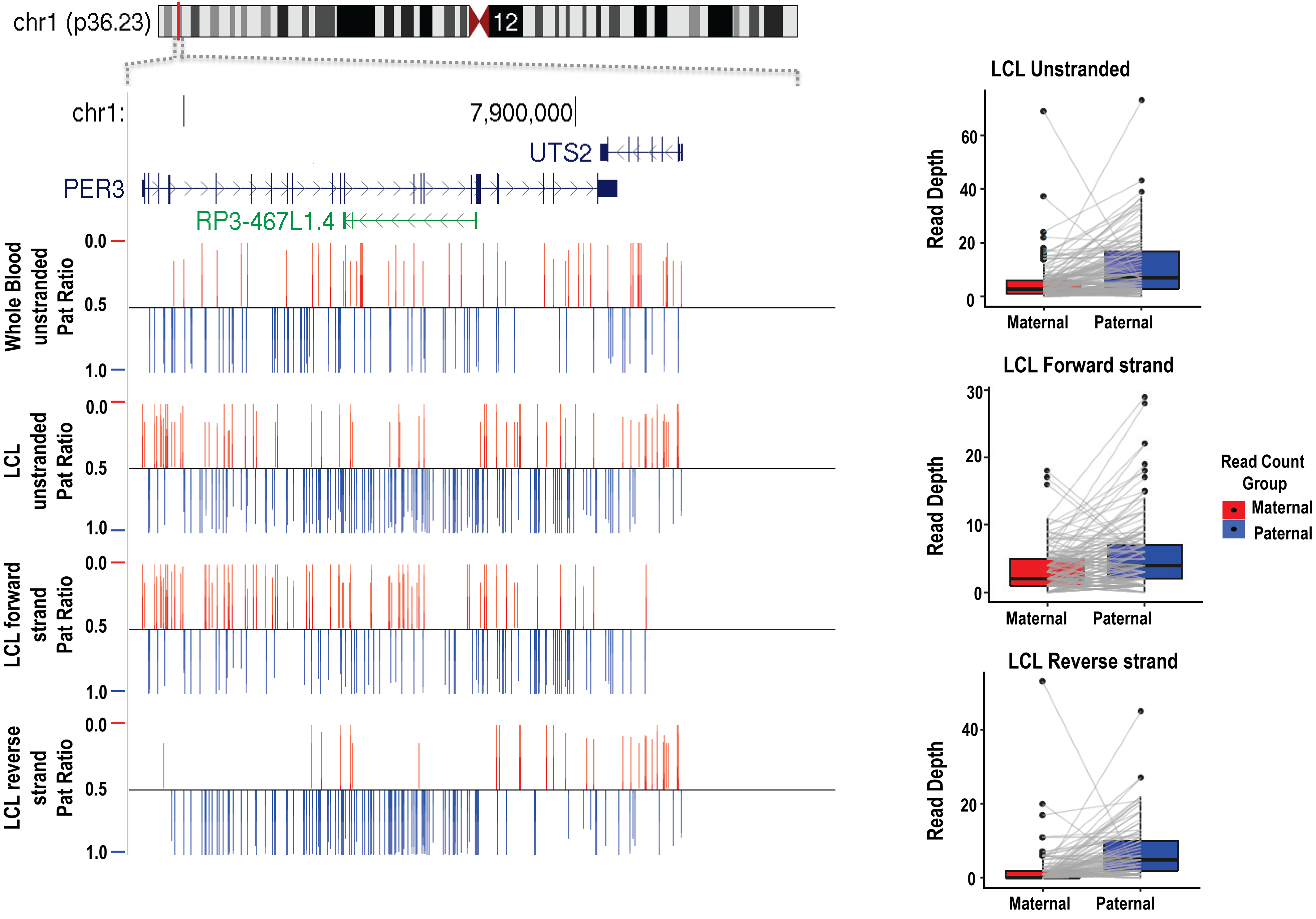

Supplement: Supplementary file 11 — Example of strand-specific data showing paternal expression bias at PER3/RP3-467L1.4 locus. PER3 and RP3-467L1.4 are two overlapping genes transcribed from opposite strands. PER3 shows incomplete imprinting, whereas RP3-467L1 shows stronger paternal bias in LCLs (paternal ratios = 0.61 and 0.81, respectively). (TIF 2840 kb) [file 12915_2019_674_MOESM11_ESM.tif]

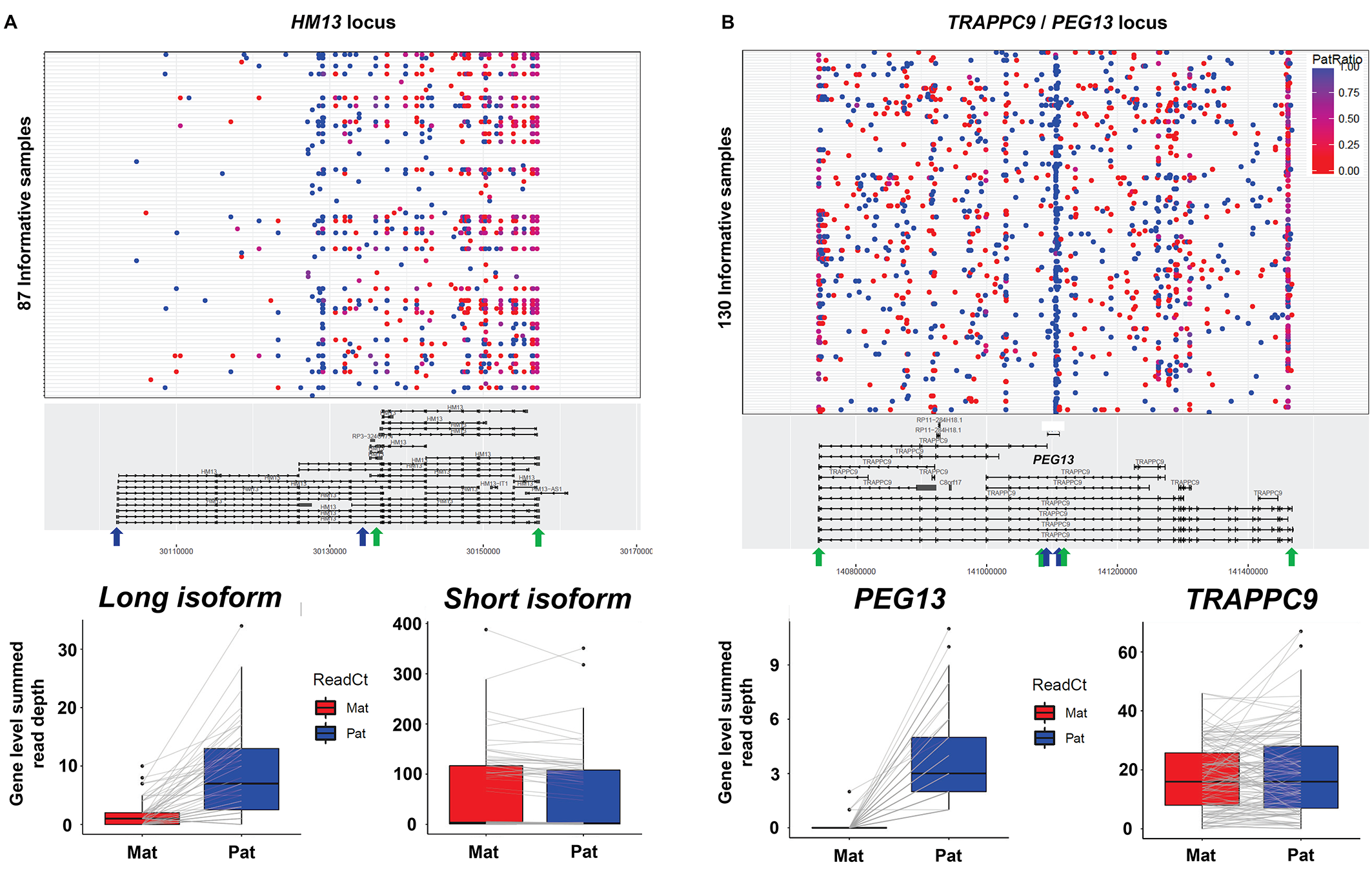

Supplement: Supplementary file 12 — Complex imprinting at HM13 and the TRAPPC9-PEG13 locus. (A) The longest isoform of HM13 shows paternal expression bias, while shorter isoforms are apparently biallelically expressed. (B) PEG13 shows exclusive paternal expression, while TRAPCC9 is biallelic. (TIF 1281 kb) [file 12915_2019_674_MOESM12_ESM.tif]

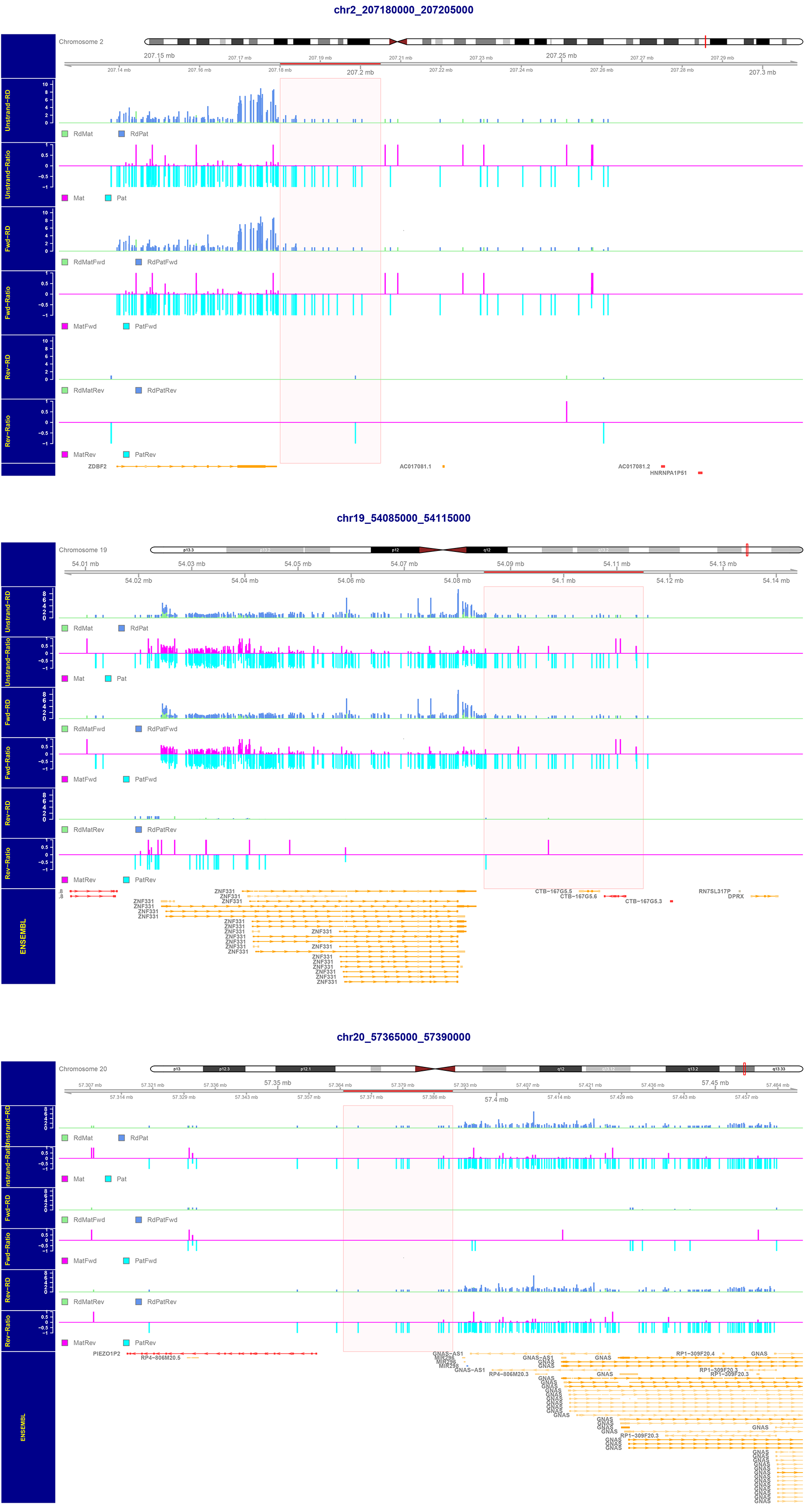

Supplement: Supplementary file 14 — Examples of significant signals of imprinting that extend beyond the annotated boundaries of genes. Possible transcriptional read-through beyond gene annotations at ZNF331, ZDBF2, and GNAS-AS1 locus. (TIF 807 kb) [file 12915_2019_674_MOESM14_ESM.tif]

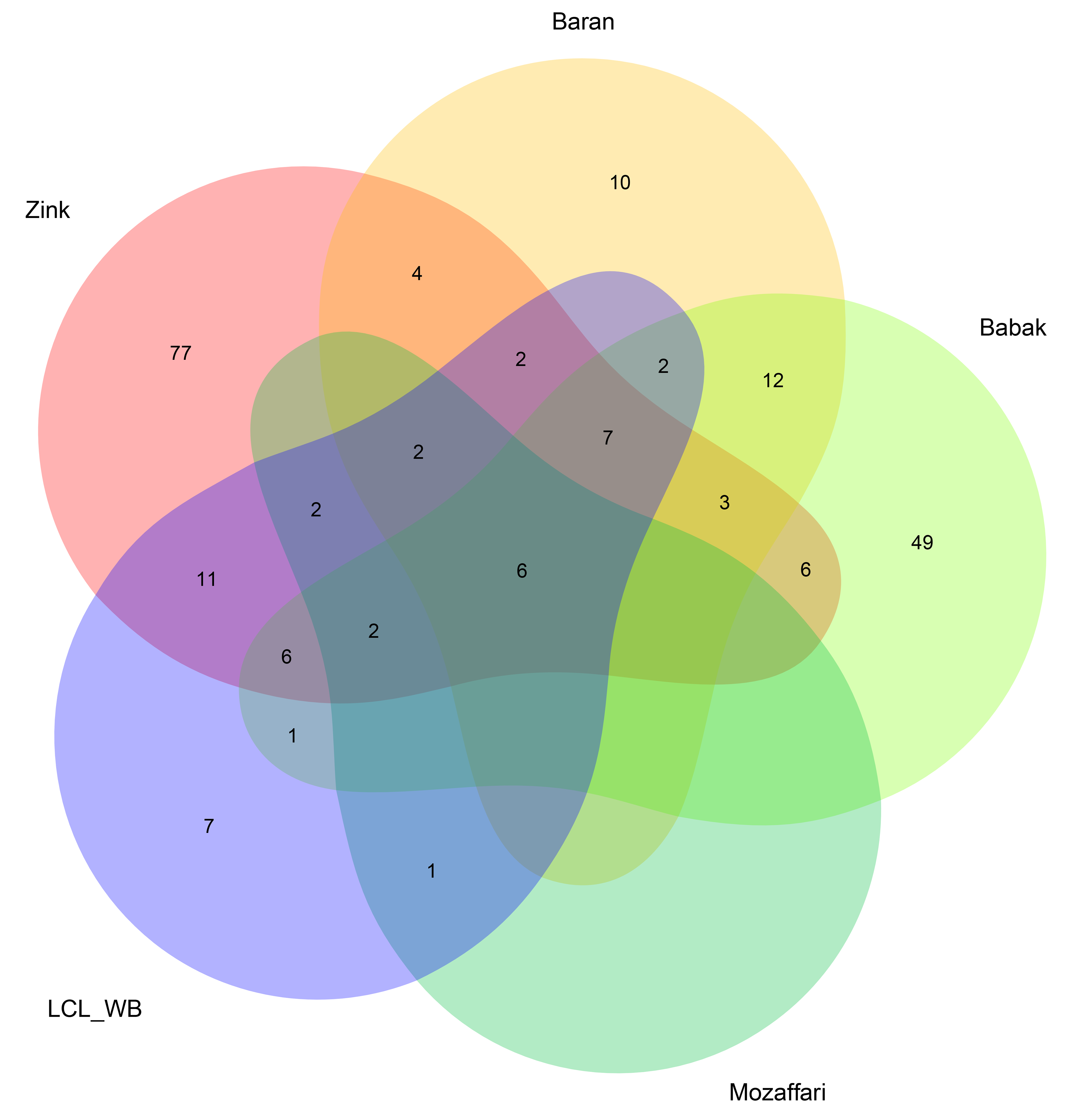

Supplement: Supplementary file 16 — Venn diagram showing an overlap of imprinted genes reported by five recent studies that utilized RNA-Seq. (TIF 657 kb) [file 12915_2019_674_MOESM16_ESM.tif]

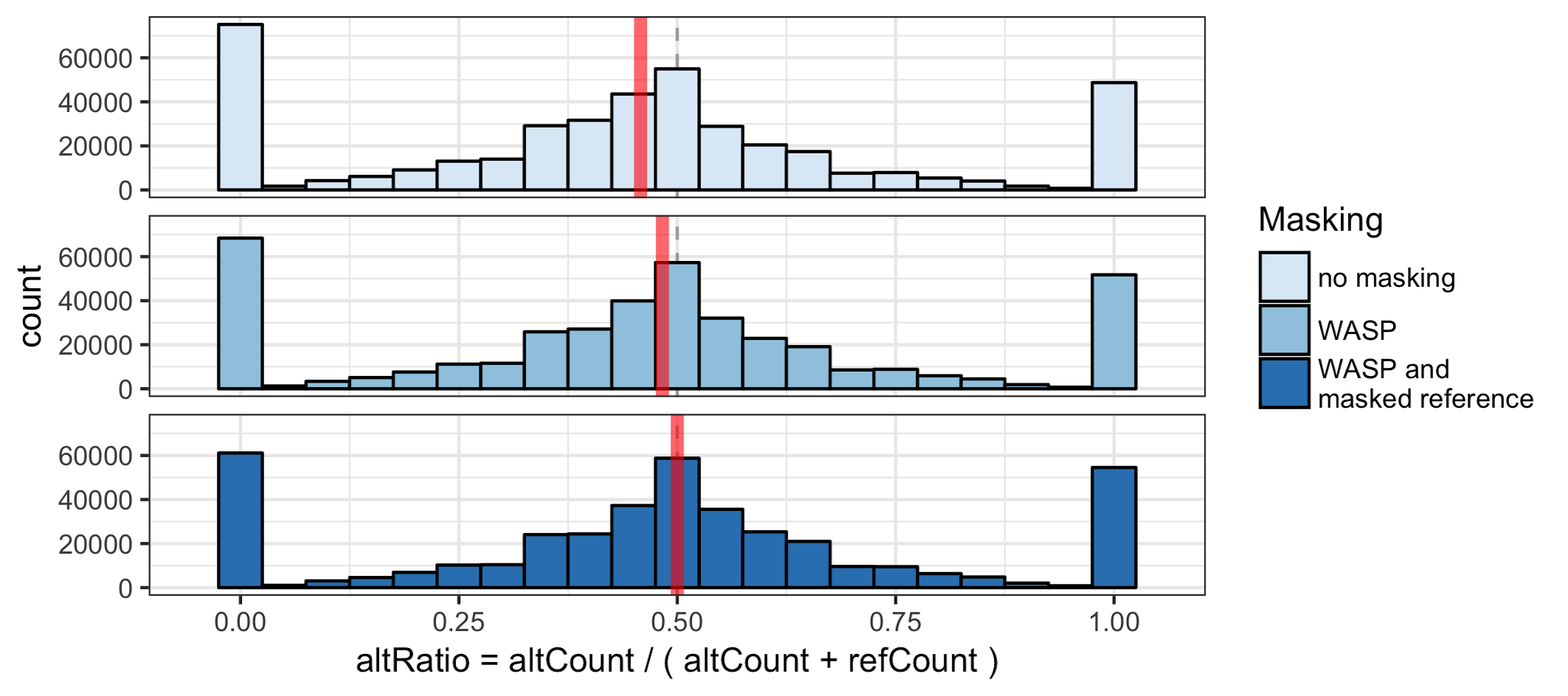

Supplement: Supplementary file 19 — The effect of masking SNV positions and utilizing WASP on reference genome mapping bias. Utilizing an unmasked reference genome, the median alternate ratio was 0.458. This increased to 0.483 after masking common SNV positions (SNVs with MAF > 0.01 were replaced by “N”) and further increased to the theoretical expectation of 0.5 after utilizing WASP to remove reads with ambiguous mapping positions. (TIF 312 kb) [file 12915_2019_674_MOESM19_ESM.tif]

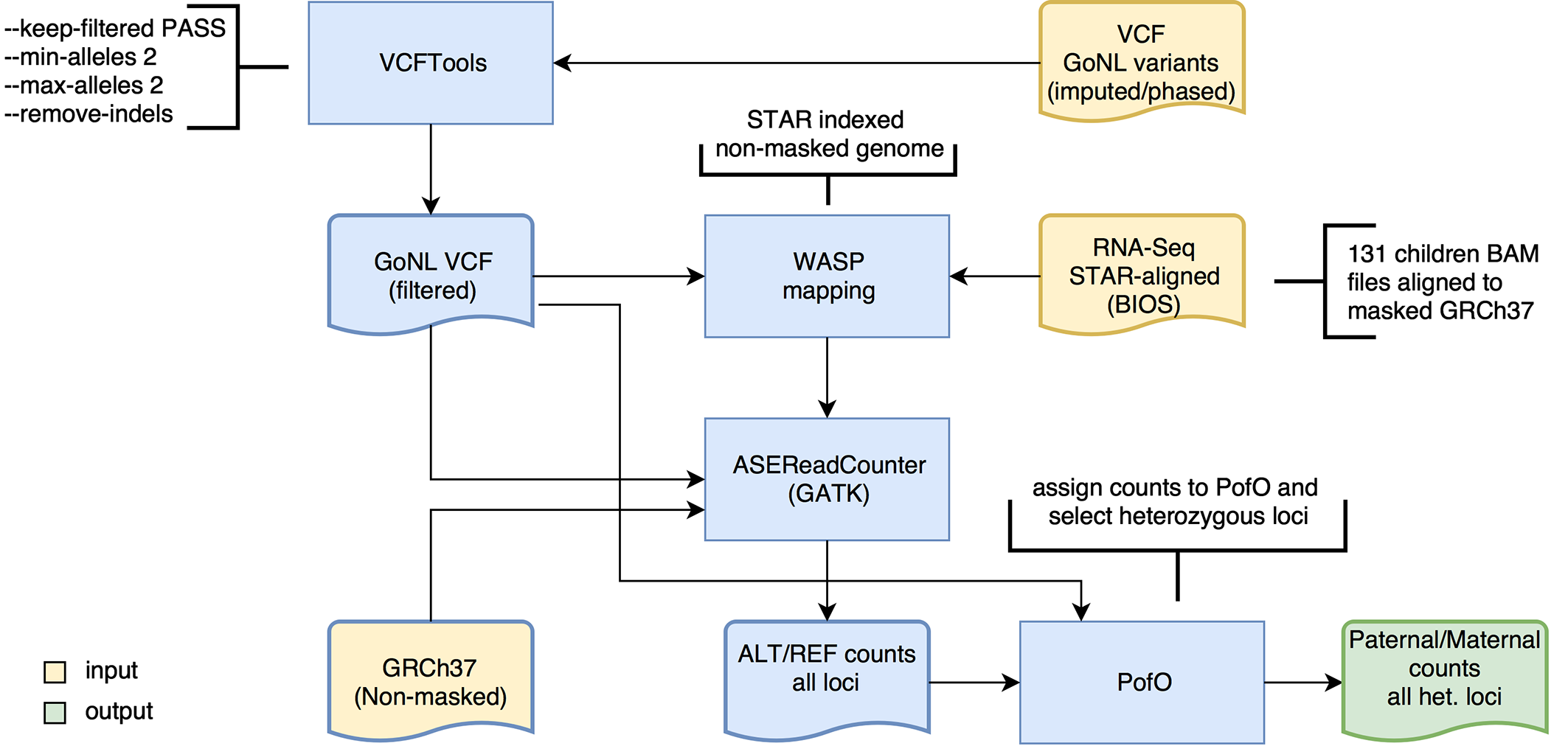

Supplement: Supplementary file 20 — A summary of the analytical pipeline used for identifying parental bias in gene expression in whole blood samples. (TIF 408 kb) [file 12915_2019_674_MOESM20_ESM.tif]

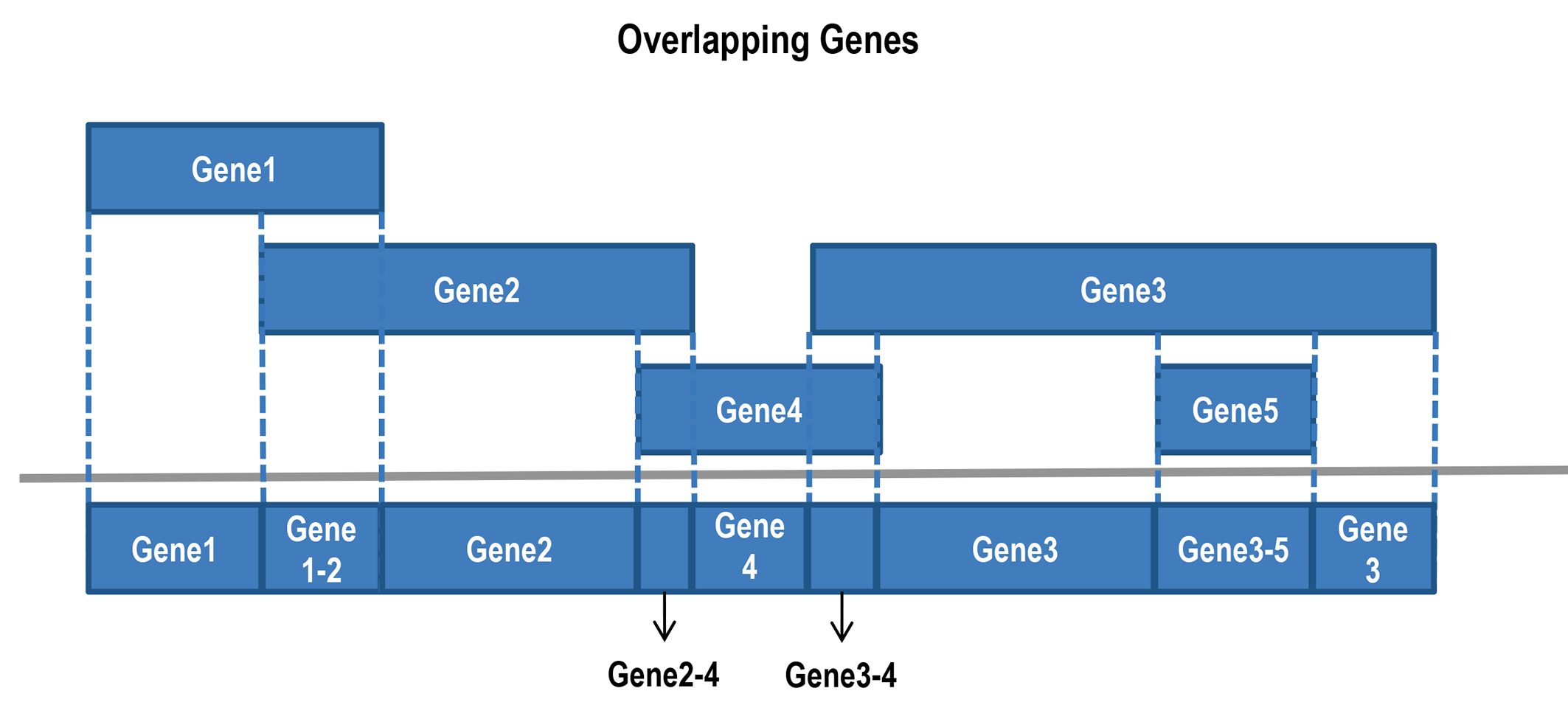

Supplement: Supplementary file 21 — Definition of unique gene fragments (UGFs). In order to avoid misassignment of reads at SNVs located within overlapping transcripts during our gene-centric analysis, we compiled all genes from Gencode annotations into a gene model where we consider overlapping regions of different genes as a separate gene. We termed these annotations unique gene fragments (UGFs). Statistical testing on each UGF was performed, and after all significant associations were compiled (Additional files 3 and 4), we manually curated each signal and removed redundant annotations, reporting a final list of 45 imprinted genes (Tables 1 and 2). (TIF 210 kb) [file 12915_2019_674_MOESM21_ESM.tif]
