# Supplementary material for: RNA-Seq in 296 phased trios provides a high-resolution map of genomic imprinting
Source: BMC Biol. 2019 Jun 24;17:50. doi: 10.1186/s12915-019-0674-0 (PMC6589892; doi:10.1186/s12915-019-0674-0)
Supplement: Supplementary file 15 — Analysis of potential age and gender effects on imprinting. (PDF 111 kb) [file 12915_2019_674_MOESM15_ESM.pdf]

Testing for age and gender effects on imprinting

We tested genes with significant parental expression bias further for possible age and gender effects. We performed three separate analyses of:

- age_WB: Age effect in WB (GoNL) samples (age information was available only for a subset of WB samples);
- gdr_LCL: Gender effect in LCL;
- gdr_WB: Gender effect in WB.

Paternal/maternal counts were aggregated per gene, as in the main analysis, and were used to calculate the PatRatio for each gene unit and sample. Genes with <5 informative samples were removed. Additionally, for gender effect analysis, genes were also removed when there were observations only from samples of one gender.

For age effect analysis a model of (PatRatio - 0.5) as a linear function of subject age was studied for each gene. For gender effect analysis the Wilcoxon Signed Rank test was used to compare average PatRatio between daughters and sons for each gene. Finally FDR correction was performed.

The table below lists the three genes with the lowest p-values for each analysis. At the FDR threshold of q<0.1, only one gene (*L3MBTL1*) was identified as putatively significant in LCL gender analysis. However, manual curation showed that this was as a false positive signal caused by a small number of outlier data points.

Analysis	Sgn	Rank	Gene unit	P-value	FDR
age_WB	FALSE	1	*MEG3,RP11-123M6.2*	0.0857	0.8279
age_WB	FALSE	2	*AC132217.4,IGF2*	0.1007	0.8279
age_WB	FALSE	3	*RP11-69E11.4,BMP8A*	0.101	0.8279
**gdr_LCL**	**TRUE**	**1**	***L3MBTL1***	**0.0007**	**0.0442**
gdr_LCL	FALSE	2	*PEG10*	0.042	0.5858
gdr_LCL	FALSE	3	*SGK2*	0.0439	0.5858
gdr_WB	FALSE	1	*MSH2*	0.0194	0.4518
gdr_WB	FALSE	2	*RP11-69E11.4,BMP8A*	0.0312	0.4518
gdr_WB	FALSE	3	*NHP2L1*	0.0357	0.4518
