# Supplementary material for: RNA-Seq in 296 phased trios provides a high-resolution map of genomic imprinting
Source: BMC Biol. 2019 Jun 24;17:50. doi: 10.1186/s12915-019-0674-0 (PMC6589892; doi:10.1186/s12915-019-0674-0)
Supplement: Supplementary file 23 — Members of the Genome of the Netherlands (GoNL) Consortium. (PDF 43 kb) [file 12915_2019_674_MOESM23_ESM.pdf]

Genome of the Netherlands Consortium:

Steering group. Cisca Wijmenga[1,2] (principal investigator), Morris A. Swertz[1–3], P. Eline Slagboom[4], Gert-Jan B. van Ommen[5], Cornelia M. van Duijn[6], Dorret I. Boomsma[7], Paul I.W. de Bakker[8–11]

Ethical, legal, and social issues. Jasper A. Bovenberg[12]

Cohort collection and sample management. P. Eline Slagboom[4], Anton J.M. de Craen[4], Marian Beekman[4], Albert Hofman[6], Dorret I. Boomsma[7], Gonneke Willemsen[7], Bruce Wolffenbuttel[13], Mathieu Platteel[1]

Sequencing. Yuanping Du[14], Ruoyan Chen[14], Hongzhi Cao[14], Rui Cao[14], Yushen Sun[14], Jeremy Sujie Cao[14]

Analysis group. Morris A. Swertz[1–3] (Co-Chair), Freerk van Dijk[1,2], Pieter B.T. Neerincx[1,2], Patrick Deelen[1,2], Martijn Dijkstra[1,2], George Byelas[1,2], Alexandros Kanterakis[1,2], Jan Bot[15], Kai Ye[4], Eric-Wubbo Lameijer[4], Martijn Vermaat[3,5,16], Jeroen F.J. Laros[3,5,16], Johan T. den Dunnen[5,16], Peter de Knijff[5], Lennart C. Karssen[6], Elisa M. van Leeuwen[6], Najaf Amin[6], Vyacheslav Koval[17], Fernando Rivadeneira[17], Karol Estrada[17], Jayne Y. Hehir-Kwa[18], Joep de Ligt[18], Abdel Abdellaoui[7], Jouke-Jan Hottenga[7], V. Mathijs Kattenberg[3,7], David van Enckevort[3], Hailiang Mei[3], Mark Santcroos[19], Barbera D.C. van Schaik[19], Robert E. Handsaker[11,20], Steven A. McCarroll[11,20], Evan E. Eichler[21], Arthur Ko[21], Peter Sudmant[21], Laurent C. Francioli[8], Wigard P. Kloosterman[8], Isaac J. Nijman[8], Victor Guryev[22], Paul I.W. de Bakker[8–11] (Co-Chair)

1. Department of Genetics, University Medical Center Groningen and University of Groningen, Groningen, The Netherlands

2. Genomics Coordination Center, University Medical Center Groningen and University of Groningen, Groningen, The Netherlands

3. Netherlands Bioinformatics Centre, Nijmegen, The Netherlands

4. Section Molecular Epidemiology, Department of Medical Statistics and Bioinformatics, Leiden University Medical Center, Leiden, The Netherlands

5. Department of Human Genetics, Leiden University Medical Center, Leiden, The Netherlands

6. Department of Epidemiology, Erasmus Medical Center, Rotterdam, The Netherlands

7. Department of Biological Psychology, VU University, Amsterdam, The Netherlands

8. Department of Medical Genetics, University Medical Center Utrecht, Utrecht, The Netherlands

9. Department of Epidemiology, University Medical Center Utrecht, Utrecht, The Netherlands

10. Division of Genetics, Brigham and Women's Hospital, Harvard Medical School, Boston, Massachusetts

11. Broad Institute of Harvard and MIT, Cambridge, Massachusetts

12. Legal Pathways Institute for Health and Bio Law, Aerdenhout, The Netherlands

13. Department of Endocrinology, University Medical Center Groningen, Groningen, The Netherlands

14. BGI, Shenzhen, China

15. Leiden Institute of Advanced Computer Science, Leiden University, Leiden, The Netherlands

16. Center for Human and Clinical Genetics and Leiden Genome Technology Center, Leiden University, Leiden, The Netherlands

17. Department of Internal Medicine, Erasmus Medical Center, Rotterdam, The Netherlands

18. Department of Human Genetics, Radboud University Nijmegen Medical Centre, Nijmegen, The Netherlands

19. Bioinformatics Laboratory, Department of Clinical Epidemiology, Biostatistics and Bioinformatics, Amsterdam Medical Center, Amsterdam, The Netherlands

20. Department of Genetics, Harvard Medical School, Boston, Massachusetts

21. Department of Genome Sciences, University of Washington, Seattle, Washington

22. Hubrecht Institute, Utrecht, The Netherlands
